# Blood-brain barrier failure as a core mechanism in cerebral small vessel disease and dementia: evidence from a cohort study

**DOI:** 10.1016/j.jalz.2016.09.006

**Published:** 2017-06

**Authors:** Joanna M. Wardlaw, Stephen J. Makin, Maria C. Valdés Hernández, Paul A. Armitage, Anna K. Heye, Francesca M. Chappell, Susana Muñoz-Maniega, Eleni Sakka, Kirsten Shuler, Martin S. Dennis, Michael J. Thrippleton

**Affiliations:** a Centre for Clinical Brain Sciences, University of Edinburgh, Edinburgh, UK; b Academic Unit of Radiology, Department of Cardiovascular Science, University of Sheffield, Royal Hallamshire Hospital, Sheffield, UK

**Keywords:** Blood brain barrier, Small vessel disease, Stroke, White matter hyperintensities, Dementia

## Abstract

**Introduction:**

Small vessel disease (SVD) is a common contributor to dementia. Subtle blood-brain barrier (BBB) leakage may be important in SVD-induced brain damage.

**Methods:**

We assessed imaging, clinical variables, and cognition in patients with mild (i.e., nondisabling) ischemic lacunar or cortical stroke. We analyzed BBB leakage, interstitial fluid, and white matter integrity using multimodal tissue-specific spatial analysis around white matter hyperintensities (WMH). We assessed predictors of 1 year cognition, recurrent stroke, and dependency.

**Results:**

In 201 patients, median age 67 (range 34–97), BBB leakage, and interstitial fluid were higher in WMH than normal-appearing white matter; leakage in normal-appearing white matter increased with proximity to WMH (*P* < .0001), with WMH severity (*P* = .033), age (*P* = .03), and hypertension (*P* < .0001). BBB leakage in WMH predicted declining cognition at 1 year.

**Discussion:**

BBB leakage increases in normal-appearing white matter with WMH and predicts worsening cognition. Interventions to reduce BBB leakage may prevent SVD-associated dementia.

## Highlight

• In 201 patients with non-disabling stroke and white matter hyperintensities (WMH), we measured blood-brain barrier (BBB) leakage with contrast-enhanced MRI.
• BBB leakage was higher in WMH than in normal appearing white matter.
• BBB leakage increased in both WMH and normal appearing white matter with the burden of small vessel disease, hypertension, and age.
• BBB leakage predicted cognitive decline one year later.
• The leakage pattern supported the hypothesis that BBB leak was pathogenic of diffuse brain damage in cerebral small vessel disease.

## 1 Introduction

Worldwide, 36 million people are estimated to be living with dementia [1]. Cerebral small vessel disease (SVD) causes about 40% of these dementias, alone or in mixed pathologies [2]. SVD also causes a fifth of the 15 million strokes that occur per year worldwide [1]. These three-million SVD (or lacunar) strokes are not severe; so, most lacunar stroke patients survive physically independent, but 36% have mild cognitive impairment or dementia [3]. The frequent cognitive impairment may reflect the association of lacunar stroke with other SVD features [4]. These features (white matter hyperintensities [WMH], lacunes, and microbleeds) are typically regarded as clinically “silent” but substantially increase the risk of dementia and stroke individually [5], [6], [7] and combined predict cognitive impairment [8].

Alzheimer's disease (AD) and stroke are typically managed and researched separately, but there is substantial evidence of overlap in pathogenesis, for example, dementia expression in life closely reflects the burden of microvascular disease more than of typical AD pathology (amyloid β plaques and neurofibrillary tangles) at postmortem [9], [10], [11]. WMH are common in AD [12]. Management of vascular risk factors [13], lifestyle interventions [14], and stroke prevention [15] could help prevent dementia. However, direct application of vascular prevention strategies that are effective in preventing large artery atherothromboembolic stroke may be ineffective or hazardous if given long term to patients with SVD or AD. Thus, blood pressure reduction and dual antiplatelet drugs failed to prevent cognitive decline or recurrent stroke [16], dual versus single antiplatelet drugs were hazardous after lacunar stroke [17], and antiplatelet drugs increased risk of cerebral hemorrhage in AD [18], reflecting our incomplete understanding of mechanisms underlying SVD and AD [19], [20], and that a different approach is needed [21].

A potential contributor to, or initiator of, the microvascular damage common to both SVD and AD is cerebral microvessel endothelial (or blood-brain barrier [BBB]) failure [22], [23], [2]. This could explain the perivascular cell and protein infiltrates, perivascular edema, and secondary axonal and neuronal damage seen pathologically in sporadic SVD [24], [25]. It could also provide a route for entry of amyloid β and inflammatory cells into the brain in AD [26], [27]. Human studies, mostly using cerebrospinal fluid (CSF)/plasma albumin ratio, show that BBB leakage increases subtly with advancing age and is worse in dementia (including AD) than in age-matched controls [28]. The BBB is also more leaky in white and deep gray matter in diabetes-associated mild cognitive impairment [29], in white matter and CSF in lacunar than atherothromboembolic stroke [30], and in white matter in patients with leukoaraiosis [31], vascular [32], and Alzheimer's dementias [33], [34]. Recently BBB leakiness was noted to increase in the hippocampus (but not other tissues) with mild cognitive impairment [35].

These studies of BBB function in vivo in patients to date have been small (all *n* < 50 except 1 [30]) sampled small volumes of brain [35] or used permeability models that ignore aging effects on blood volume and vascular surface area [36], [37] that limit the measurement of permeability accurately. Thus, there is no comprehensive, whole-brain, tissue-specific, in vivo assessment of BBB leakiness in human SVD and none with concurrent independent measures of brain interstitial fluid or tissue damage, making it unclear if BBB leakage is real, pathogenic, or an epiphenomenon in SVD. If pathogenic, then we hypothesized that the leak should worsen with worsening SVD burden, be spatially related to major markers of SVD such as WMH, and be accompanied by increased interstitial fluid. BBB leakage should increase with age, in small vessel (i.e., lacunar) versus atherothromboembolic (i.e., cortical or large artery) ischemic stroke [30] and in hypertension (a major SVD risk factor [38]), and predicts worsening of SVD-associated clinical or imaging features.

We prospectively studied a large cohort of patients with lacunar stroke (a model for vascular effects on neurodegeneration that identifies patients at high risk of cognitive impairment [3]) and cortical ischemic stroke (a control group with similar vascular risk factors [39] and medications), with a range of WMH, followed up at 1 year. We examined the magnitude and spatial distribution of BBB leak and tissue integrity in relation to WMH as a major marker of SVD, using three-dimensional (3D), whole-brain, dynamic contrast-enhanced (DCE) magnetic resonance imaging (MRI), diffusion-tensor imaging, T1 mapping, and spatially detailed, tissue-specific analysis.

## 2 Methods

### 2.1 Recruitment and eligibility

**Fig. 1:**
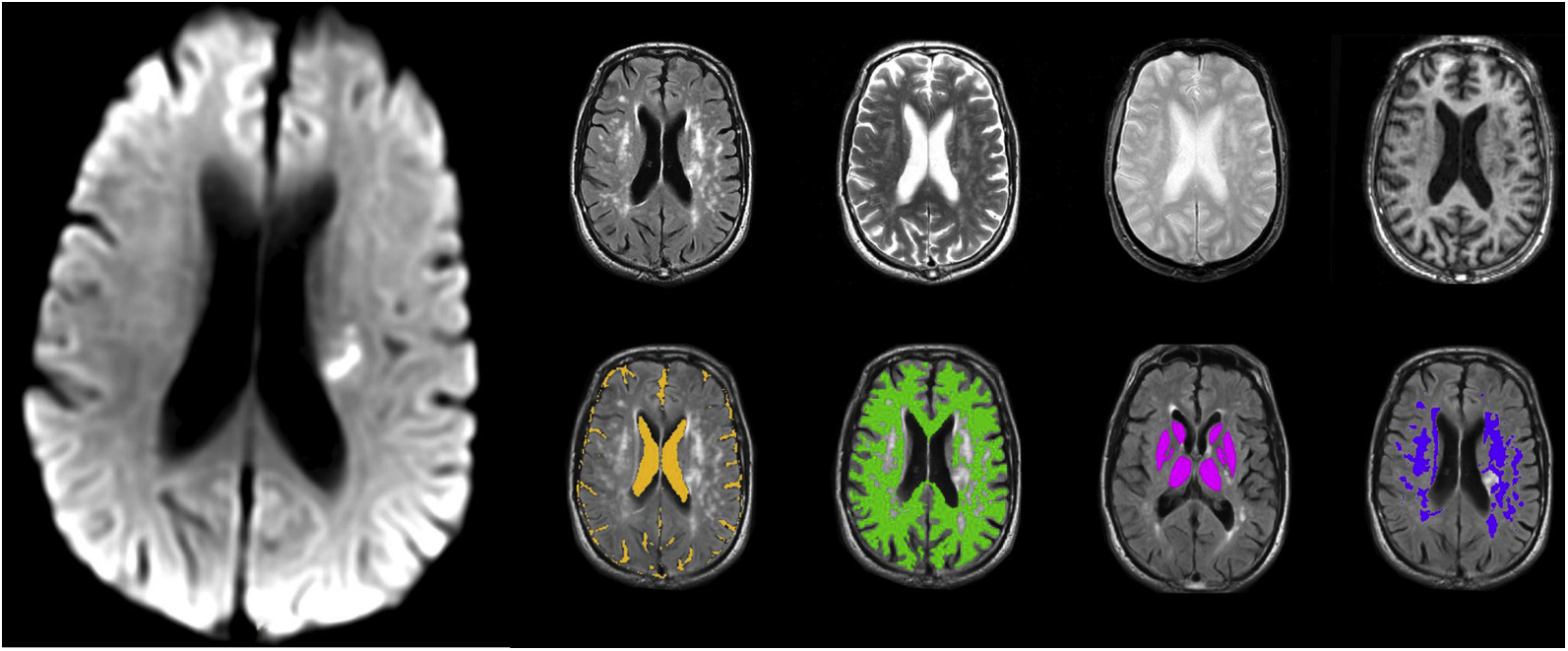
Magnetic resonance brain imaging sequences and processed images. Left, diffusion-weighted image shows recent small deep infarct in the left centrum semiovale (white area) as the index infarct. Top row, left to right, fluid-attenuated inversion recovery and T2-, T2*-, and T1-weighted axial brain images at the same level as in the large diffusion-weighted image on the left. Bottom row, left to right, colors indicated masking obtained by semiautomated image processing to identify cerebrospinal fluid (yellow), normal-appearing white matter (green), deep gray matter (pink), and white matter hyperintensities (purple); note the index infarct was masked by hand.

We recruited patients prospectively who presented with a lacunar or mild cortical ischemic stroke classified clinically using the risk-factor-free Oxfordshire Community Stroke Project classification [40]. We included patients aged ≥18 years, able to consent, within 4 weeks of mild ischemic stroke (i.e., National Institutes of Stroke Scale score [NIHSS] ≤5, unlikely to cause physical dependency), with an MR diffusion-weighted imaging (DWI) infarct compatible with the index stroke symptoms (Fig. 1), or no other cause of symptoms, and no life-threatening illness to preclude 1 year follow-up. We excluded patients who were unable to tolerate MRI or gadolinium-containing intravenous contrast agent.

### 2.2 Ethics

Lothian Ethics of Medical Research Committee (REC 09/81,101/54) and NHS Lothian R+D Office (2009/W/NEU/14) approved the study. All patients gave written informed consent.

### 2.3 Clinical assessments and outcomes

A specialist stroke physician recorded the medical history and examination including stroke severity (NIHSS, for details, see Supplementary Material). A panel of stroke experts assigned the stroke subtype (lacunar or cortical) using the clinical syndrome [40] and acute infarct appearance on DWI MRI. In case of discrepancies, the imaging subtype was used.

We introduced cognitive testing after the study had started and assessed current cognition in as many patients as possible thereafter (Addenbrooke Cognitive Assessment—Revised [ACE-R]), premorbid intelligence (National Adult Reading Test [NART]), and depression (Beck Depression Index) at 1–3 months after stroke concurrent with BBB imaging (Section 2.4 below).

At 1 year, we assessed all patients for dependency (Oxford Handicap Scale [41], similar to the modified Rankin Scale), recurrent vascular events, cognition (ACE-R), and repeated MRI for WMH burden, new infarcts, or hemorrhages. Patients unable to attend were followed up remotely.

### 2.4 Neuroimaging

All MR examinations were performed on one 1.5-T GE Signa HDxt scanner, with tight quality assurance. Diagnostic MR included T1, T2, T2*, and DTI sequences (see “Online Methods” and Supplementary Table 1 in Supplementary Material [42]) to assess infarcts and SVD features [43]. We performed DCE MRI [37] for BBB leak at 1–3 months after stroke (to minimize the index stroke effect on BBB) and T1 mapping for brain water content (see Supplementary Material). After two 3D fast-spoiled gradient-echo acquisitions (flip angles 2 and 12°) for precontrast T1 (T_10_) maps, we injected gadoterate meglumine (Gd-DOTA, DOTAREM; Guerbet, Paris, France) 0.2 mL/kg (i.e., 0.1 mmol/kg body weight) at 2 mL/second intravenously via injection pump and then repeated the 3D T1-weighted sequence sequentially 20 times for 24 minutes [44], [36], [37], using long acquisition times to detect subtle BBB leak [36], [35].

### 2.5 Image processing

We analyzed all imaging data blind to clinical and other imaging information using validated, qualitative, and quantitative assessments (see “Online Methods” in Supplementary Material [42]), as previously [45]. We identified the index infarct and SVD lesions using visual scores (see “Online Methods” in Supplementary Material). On coregistered images, we separated CSF, whole-brain, WMH, and normal-appearing white and gray matter [46], [47] (Fig. 1), differentiating WMH into “more” and “less” intense by degree of abnormality on fluid-attenuated inversion recovery and T2 and T1 sequences. For spatial analysis of BBB leak distribution, we also divided normal-appearing white matter into 10 “contours” each two voxels (≈2 mm) wide from the WMH edge outward (piloted in [48]). We extracted signal intensities from the DCE-MRI pre-post contrast curves per tissue, per voxel, and per time point after intravenous contrast (see “Online Methods” in Supplementary Material) [44]. We used sagittal sinus to correct for intravascular contrast [44] as carotid arteries have significant limitations especially in older subjects [37]. We calculated precontrast T1 (longitudinal relaxation time, T_10_ [44], milliseconds) to control for precontrast tissue characteristics [36], [44].

### 2.6 Sample size

For 80% power, a two-sided test, the estimated sample size was 170 patients to reach 1 year follow-up. Allowing for about 10% dropout required 200 patients to have BBB imaging (see “Online Methods” in Supplementary Material).

### 2.7 Statistical analysis

**Fig. 2:**
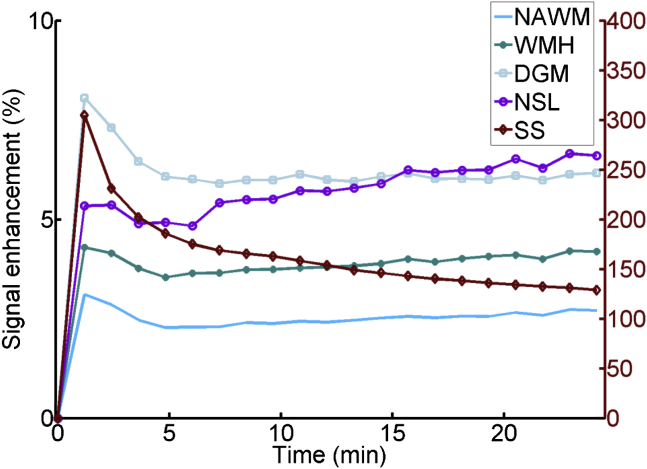
Change in signal postcontrast in different brain tissues by time averaged across all patients. The graph demonstrates that signal in the blood pool declines after the first pass of bolus but increases progressively in all brain tissues meaning that intravascular contrast does not account for the tissue signal changes. NAWM, normal appearing white matter; WMH, white matter hyperintensities; DGM, deep gray matter; NSL, new (index) stroke lesion; SS, sagittal sinus (represents the blood pool and provides an arterial input function for statistical modeling purposes).

Several mathematical models have been proposed to estimate BBB permeability [36], [37]. The Patlak method best suits low permeability states [36], [49], [50], but all models rely on assumptions regarding capillary surface area that are invalid in low permeability states, for example, that capillary density and blood volume are constant, whereas both vary between tissues and decrease with age and in disease [37]. In the individual tissues and subjects, the actual capillary density is unknown, and it would introduce further potential confounds to use constant values. We explored permeability modeling methods extensively, including in simulations [44], [37], tried to obtain estimates of capillary density to provide realistic factors, but none of these were adequate for use in a wide age range and disease range population such as here. We found strong effects of age on BBB, T1, mean diffusivity (MD), and fractional anisotropy (FA) in separate analyses [47], [48]. Therefore, in our prespecified analysis (see “Protocol Online” in Supplementary Material), we did not calculate permeability but used linear mixed modeling of the signal enhancement slopes (Fig. 2) to identify tissue- and patient-specific differences in BBB leakage. All BBB analyses were adjusted for age, WMH burden, vascular risk factors, intravascular contrast, baseline tissue T1, and time after contrast injection (full statistical details, see “Online Methods” in Supplementary Material). Analysis of ACE-R also tested for interactions between lacunar and cortical subtypes. We used SAS 9.3 (www.sas.com) for all analyses and R 2.13.1 for graphs.

## 3 Results

We recruited 264 patients (Supplementary Fig. 1): 42 declined detailed MRI, 14 were not well enough for BBB imaging, and BBB or structural data were insufficient for analysis in 7, leaving 201 with complete data. The 63 patients without BBB imaging had slightly more severe strokes (median NIHSS 2, interquartile range [IQR] 2–4) than the 201 with BBB imaging (NIHSS 2, IQR 1–3, *P* = .03) and were slightly older (66 vs 69 years, *P* = .025), but there were no other differences.

The 92 of 201 (46%) patients with lacunar stroke did not differ significantly from the 109 (54%) with cortical stroke in age (mean: 64, IQR 56–72; 68, IQR 60–76, respectively, *P* = .097) proportion of men, with hypertension, diabetes, or hyperlipidemia (Supplementary Table 2). Lacunar strokes were more severe than cortical strokes (NIHSS medians: 1, IQR 0–2; vs 1, IQR 0–1, respectively, *P* = .002), with no difference in time to initial assessment (medians: 3, 1–6; vs 3, 1–8 days, respectively, *P* = .71). Cortical patients had more embolic sources than lacunar (25.7% vs 12%, *P* = .014). Cognitive testing was available for 147 (73%) at 1–3 months (median ACE-R, 90, IQR 83–95) and in 139 (69%) at 1 year (median ACE-R 92, IQR 85–95).

Compared with normal-appearing white matter, BBB leakage was higher in WMH (*P* = .025), CSF (*P* < .0001), index (*P* < .0001), and old infarcts (*P* < .0001) and was lower in deep gray matter (*P* < .0001, Supplementary Table 3, Fig. 2). BBB leakage increased with (Table 1) age in all tissues, significantly in deep gray, CSF, and WMH, for example, in deep gray matter by 0.025 × 10^3^ (95% confidence interval [CI] 0.0023 × 10^3^, 0.049 × 10^3^, *P* = .030), per 5-year age increment; WMH burden, significantly in all tissues except index or old infarcts, for example, in normal-appearing white matter by 0.024 × 10^3^, (95% CI 0.002 × 10^3^, 0.047 × 10^3^, *P* = .033), per point increase in WMH score; and in index and old infarcts of cortical versus lacunar subtype. BBB leakage was significantly higher in lacunar than cortical subtype in CSF but in cortical than lacunar stroke in normal-appearing white matter and deep gray matter. BBB leakage also increased with (Table 2): hypertension significantly in all tissues, pulse pressure in some tissues, and with baseline tissue T1 (i.e., water content) in all tissues.

**Table 1:** BBB leakage and age, WMH burden (Fazekas score), and stroke subtype

Tissue	Variable	β coefficient × 10^3^	95% CI × 10^3^	*P*-value
Normal-appearing white matter	Age	0.016	−0.001, 0.033	.065
	Fazekas score	0.024	0.002, 0.047	.033
	Stroke subtype	−0.110	−0.19, −0.030	.005
Deep gray matter	Age	0.025	0.002, 0.049	.031
	Fazekas score	0.059	0.028, 0.09	.0002
	Stroke subtype	−0.11	−0.21, −0.0003	.049
CSF	Age	0.219	0.158, 0.28	<.0001
	Fazekas score	0.205	0.124, 0.287	<.0001
	Stroke subtype	0.363	0.082, 0.644	.011
WMH	Age	0.059	0.035, 0.082	<.0001
	Fazekas score	0.104	0.072, 0.136	<.0001
	Stroke subtype	−0.040	−0.150, 0.068	.46
Index infarct	Age	0.045	0.001, 0.089	.045
	Fazekas score	0.017	−0.040, 0.075	.57
	Stroke subtype	−0.520	−0.720, −0.330	<.0001
Old infarct	Age	0.049	−0.0015, 0.10	.057
	Fazekas score	0.041	−0.030, 0.11	.24
	Stroke subtype	−0.430	−0.680, −0.190	.0004

Abbreviations: BBB, blood-brain barrier; WMH, white matter hyperintensities; CI, confidence interval; CSF, cerebrospinal fluid.

NOTE. Values are BBB leakage (change in postcontrast signal per minute) per 5-year increase in age, per point increase in Fazekas score, or lacunar versus cortical stroke. The interaction coefficients for age, Fazekas score, and subtype are derived from three separate models (the data were already adjusted for key predictors listed previously and simultaneous fitting of multiple interaction terms was not supported). Age, Fazekas score, and stroke subtype are each adjusted for each other and sagittal sinus signal, brain tissue signal precontrast (T_10_), mean arterial blood pressure, diagnosis of hypertension, pulse pressure, and smoker status. In stroke subtype, a negative effect estimate indicates that values are lower in patients with lacunar than with cortical stroke. A higher Fazekas score indicates more WMH.

**Table 2:** Association between BBB leakage, vascular risk factors, and brain parameters: hypertension, mean arterial pressure, smoker status, brain tissue T1 signal precontrast, and intravascular signal (sagittal sinus), adjusted for one another, age, and combined Fazekas score

Tissue	Variable	β coefficient × 10^3^	95% CI × 10^3^	*P*-value
Normal-appearing white matter	Hypertension	0.206	0.118, 0.295	<.0001
	Mean arterial pressure	−0.00105	−0.0036, 0.0015	.41
	Pulse pressure	0.00121	−0.00061, 0.0030	.19
	Smoking status	−0.050	−0.130, 0.026	.18
	Tissue T1	0.46	0.153, 0.768	.0033
	Intravascular contrast	0.050	−0.080, 0.181	.45
Deep gray matter	Hypertension	0.233	0.111, 0.354	.0002
	Mean arterial pressure	0.00017	−0.0033, 0.0036	.93
	Pulse pressure	0.00380	0.0013, 0.0063	.003
	Smoking status	0.035	−0.080, 0.146	.53
	Tissue T1	0.365	−0.0018, 0.732	.051
	Intravascular contrast	0.00062	−0.180, 0.182	.99
CSF	Hypertension	0.338	0.016, 0.66	.040
	Mean arterial pressure	0.016	0.0064, 0.025	.0009
	Pulse pressure	0.017	0.011, 0.024	<.0001
	Smoking status	−0.30	−0.590, −0.010	.042
	Tissue T1	1.107	0.903, 1.31	<.0001
	Intravascular contrast	1.363	0.878, 1.848	<.0001
WMH	Hypertension	0.256	0.131, 0.38	<.0001
	Mean arterial pressure	0.000368	−0.0032, 0.0039	.84
	Pulse pressure	0.00333	0.00076, 0.00589	.011
	Smoking status	−0.110	−0.23, 0.0002	.050
	Tissue T1	0.536	0.163, 0.909	.0049
	Intravascular contrast	0.237	0.052, 0.423	.012
Index infarct	Hypertension	0.677	0.444, 0.91	<.0001
	Mean arterial pressure	−0.00298	−0.009, 0.003	.35
	Pulse pressure	−0.00203	−0.0066, 0.0025	.38
	Smoking status	−0.040	−0.240, 0.154	.67
	Tissue T1	1.617	1.249, 1.984	<.0001
	Intravascular contrast	−0.040	−0.390, 0.305	.81
Old infarct	Hypertension	0.472	0.208, 0.735	.0005
	Mean arterial pressure	0.016	0.007, 0.024	.0002
	Pulse pressure	0.00261	−0.0033, 0.0085	.39
	Smoking status	−0.40	−0.64, −0.16	.001
	Tissue T1	0.773	0.317, 1.228	.001
	Intravascular contrast	0.086	−0.330, 0.499	.68

Abbreviations: BBB, blood-brain barrier; CI, confidence interval; CSF, cerebrospinal fluid; WMH, white matter hyperintensities.

**Fig. 3:**
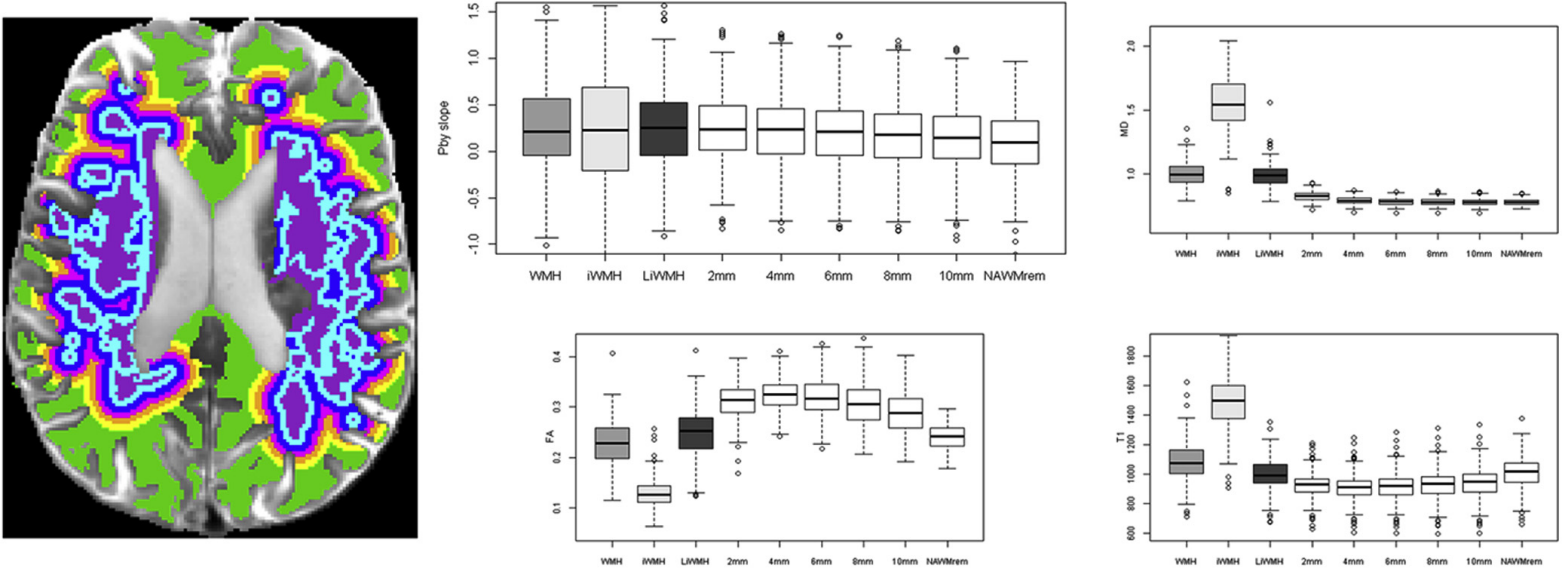
Representative axial brain magnetic resonance imaging (left, same case as in Fig. 1) showing white matter hyperintensities and contours at two voxel increments (approximately 2 mm width) extending concentrically into the normal-appearing white matter from which blood-brain barrier leakage (Pby, graph top left), mean diffusivity (graph top right, ×10^−9^ mm^2^/s), fractional anisotropy (graph bottom left), and T1 (graph bottom right, ms) were extracted. White matter hyperintensities are split into intense and less intense to determine the difference in these biomarkers by severity of white matter hyperintensities and by all white matter hyperintensities, as indicated on the x-axis. WMH, white matter hyperintensities; FA, fractional anisotropy; iWMH, intense white matter hyperintensities; liWMH, less intense white matter hyperintensities.

Analysis of spatial distribution of BBB leakage in normal-appearing white matter (Fig. 3) showed that BBB leakage increased linearly at 0.0099 × 10^3^ (95% CI 0.0114 × 10^3^, 0.0084 × 10^3^, *P* < .0001) per contour closer to the WMH edge, adjusted for age, Fazekas score, blood pressure, stroke subtype, and smoking. The leak was worst in the WMH where it was worst in the most abnormal areas (“intense” versus “less intense” WMH). Additionally, MD increased from the 4 mm contour and T1 from the 2 mm contour proximate to the WMH edge; both were yet higher in less intense and highest in intense WMH (Fig. 3), consistent with an increase in interstitial water mobility and then in measureable interstitial water close to the WMH, with the most abnormal values in the WMH. Axonal integrity (FA) decreased with increasing proximity to WMH, being lowest in the most intense WMH, consistent with most axonal disruption being in the most abnormal-looking tissue.

**Fig. 4:**
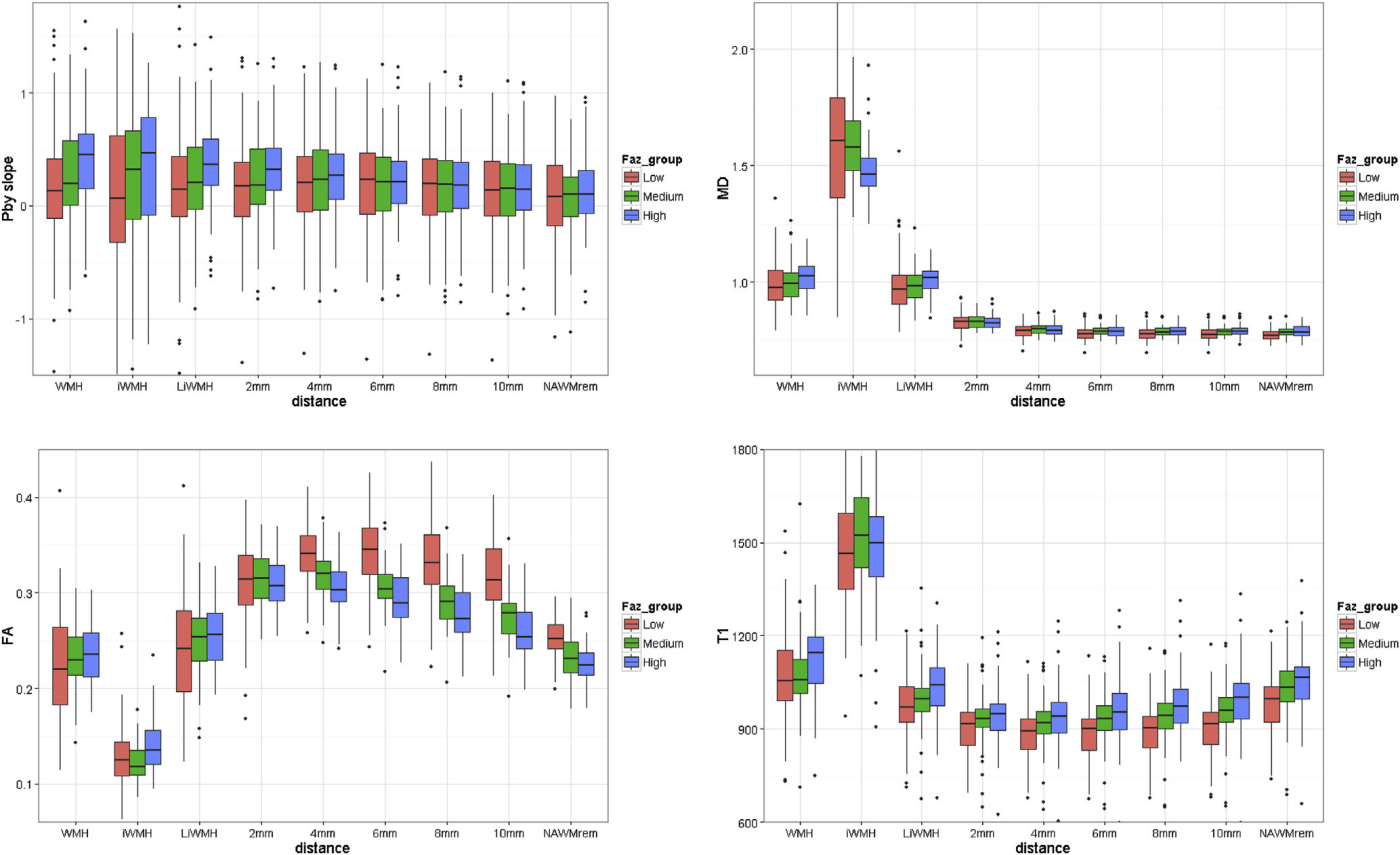
Changes in blood-brain barrier leakage (top left, Pby), mean diffusivity (top right, ×10^−9^ mm^2^/s), fractional anisotropy (bottom left), and T1 (bottom right, ms) in white matter hyperintensities and 2 mm incremental contours expanding concentrically across normal-appearing white matter, according to white matter hyperintensities burden categorized by Fazekas score into low (0–2), medium (3–4), and high (5–6) white matter hyperintensities burden. Contours as in Fig. 3, left-hand image. Supplementary Fig. 3 shows these data by individual Fazekas white matter hyperintensities scores. Data are age adjusted. Faz_group, Fazekas score group; WMH, white matter hyperintensities; MD, mean diffusivity; iWMH, intense white matter hyperintensities; LiWMH, less intense white matter hyperintensities; FA, fractional anisotropy.

On further assessing the contours by Fazekas WMH score corrected for age (Fig. 4), patients with most WMH (Fazekas score 5–6) had the highest BBB leakage, water content (MD, T1), and lowest axonal integrity (FA) in normal-appearing white matter and WMH (for details, see Supplementary Fig. 2).

At 1–3 months after stroke, ACE-R was associated negatively with age and positively with premorbid intelligence (NART), but not with WMH score or concurrent BBB leakage in any tissues, with no difference between lacunar and cortical stroke patients (Supplementary Table 4). At 1 year, BBB leakage in WMH predicted lower ACE-R in lacunar (β −3.52, 95% CI −5.9, −1.13) but not cortical (β 0.03, 95% CI −5.77, 5.12) stroke patients; high WMH score and NART, but not age, predicted ACE-R in both stroke subtypes (Supplementary Table 5). Incorporating ACE-R at 1–3 months after stroke into the 1-year ACE-R prediction model (to assess change in ACE-R and BBB leak) showed that BBB leakage in WMH predicted a decline in ACE-R at 1 year in both lacunar and cortical stroke patients (β −1.89, 95% CI −3.62, −0.16). ACE-R and NART at 1–3 months also predicted decline in ACE-R at 1 year (Supplementary Table 6) but not WMH score, hypertension, or age.

At 1 year, 21 patients had a clinically evident recurrent stroke, 19 had a new infarct on MRI (21 of 201 could not have repeat scanning, Supplementary Fig. 1), and 34 patients were dependent or dead (17%). WMH burden was the strongest predictor of recurrent stroke or dependency, overwhelming BBB associations except for BBB leakage in CSF that showed a weak association with dependency (Supplementary Table 7). Vascular risk factors did not predict outcome.

## 4 Discussion

We show, in this large stratified patient cohort, that subtle increases in BBB leakage appear to be widespread in normal-appearing white matter and more pronounced in WMH, increasing with the visible severity of tissue damage. The BBB leak is accompanied by increased interstitial water mobility and water content close to and in the WMH, providing support for the BBB leak findings. BBB leakage in normal-appearing white matter and in WMH was worst in patients with severe WMH, with hypertension and increased pulse pressure. BBB leakage in WMH predicted cognitive impairment at 1 year in lacunar stroke and cognitive decline between 1 and 3 months and 1 year after stroke in both lacunar and cortical stroke patients. Taken together, these observations suggest that BBB leakage precedes increases in interstitial fluid and axonal damage, all which worsen as WMH worsen; BBB leak in WMH also predicts cognitive decline at 1 year, particularly in lacunar stroke patients who are already known to be at high risk of cognitive decline after stroke [3]. We show that WMH associate with BBB leakage regardless of the underlying stroke subtype, findings that are generalizable to older subjects with WMH. Thus, BBB leakage appears to be important in pathogenesis of SVD-associated brain damage in large clinical populations. The association between BBB leak, hypertension, and pulse pressure provides a mechanism whereby hypertension may worsen WMH [38] and thus may contribute to declining cognition. Pathogenesis involving BBB leak may also explain the apparent lack of benefit for antithrombotic drugs and increased hemorrhage risk in SVD [17], [51] and AD [18].

We provide conclusive in vivo evidence that normal-appearing white matter, in the presence of WMH, is not normal, at any adult age, but increasingly abnormal with increasing WMH burden [48], [47] and by proximity to WMH [52], [53]. In comparison with recent work [52], our study provides more extensive spatial sampling of brain white matter and multiple independent imaging parameters. We show, in this much larger sample, that subtle BBB failure can be detected with DCE MRI in large clinical studies. In comparison with recent work [35], our study provides full brain coverage, volumetric analysis of BBB leakage across spatially related tissue while preserving sensitivity to low-grade BBB leak [37], a large, highly phenotyped, clinically relevant patient sample, and assumption-free statistical analysis. This approach was based on extensive testing of models including simulated data which made it clear that current models were not suitable for low permeability states across a wide range of age and disease [37]. The differences in BBB leakage in index and old infarcts demonstrate the sensitivity of this method to detect BBB leakage.

Why should increased BBB leakage occur in SVD and is this also relevant to AD? AD and SVD pathologies commonly co-occur, cognition is worse when SVD is present in AD [12], vascular risk factors accelerate both disorders, both are associated with elevated systemic inflammatory markers, and microglial activation is common in both diseases pathologically. The BBB has a huge surface area, handles a fifth of the cardiac output at rest, and maintains the correct brain interstitial milieu within tight limits for normal brain function, a major physiological challenge [2]. A slight decline in barrier function could result in vascular wall and perivascular parenchymal damage from fluid shifts [54] or allow neurotoxins such as serum amyloid P, an important precursor to amyloid deposition [27], into the brain. The findings are consistent with BBB failure being an early pathogenic step in SVD, with axonal loss occurring secondarily [54], [20]. BBB integrity declines with normal aging and is worse in AD according to biochemical [28] and pathological [33] analyses. BBB failure may occur through multiple factors, for example, hypertension, diabetes, inflammation [24], smoking, and increased sodium intake [50], all damage the vascular endothelium. Cerebral blood flow is reduced in both SVD and AD: hypoperfusion triggers BBB failure in experimental models, and fibrinogen leakage across the BBB was associated with astrocyte morphological changes, reversal of aquaporin 4 distribution on end feet implying glyovascular malfunction, and irreversible damage in WMH seen at postmortem in patients with cognitive impairment after stroke in life [55]. WMH heritability [56] and genetics [57] suggest that some individuals may be more susceptible to damage in the neurovascular unit, including the BBB [58], perhaps accounting for variation in SVD-related brain damage and clinical expression between individuals with apparently similar risk factor exposures.

Our results provide evidence that BBB leakage is an important, likely early pathological event in development of SVD-associated brain damage. WMH should sound alarm bells to identify potentially modifiable risk factors at all ages and in all dementias. Brain interstitial fluid shifts are potentially remediable, at least initially: reduction in interstitial fluid, for example, by improved endothelial function and reduced BBB leak, might prevent accumulation of permanent brain injury, slowing neurodegeneration, preserving cognition, and preventing dementia and stroke. Persistent BBB malfunction may precipitate worsening of microvessel wall injury, with secondary inflammation [24], impaired vasoreactivity [59], or luminal narrowing and hence tissue ischemia—a vicious cycle of brain damage. Future research should target ways to reduce small vessel endothelial damage to prevent progressive BBB breakdown and brain injury including, but not restricted to, improved management of lifestyle factors (e.g., exercise [14], diet [50]) and vascular risk factors (e.g., hypertension).

**Research in Context**

1. Systematic review: We reviewed the literature on blood-brain barrier (BBB) leakage detected with magnetic resonance imaging in humans during aging, in small vessel disease (SVD) or dementia, from multiple electronic bibliographic databases and identified six studies (*n* = 203).
2. Interpretation: Our study doubles the available data on BBB leakage and provides independent evidence on the hypothesis that BBB leakage is pathogenic in SVD-related brain damage including tissue fluid measures, spatial distribution, two SVD markers, age, disease burden, risk factor (hypertension), and cognition associations.
3. Future directions: Our results provide a testable hypothesis, a route for amyloid entry to the brain, and should encourage new thinking about SVD and/or aging-related brain damage and cognitive decline beyond ischemia or amyloid deposition. Future research should focus on the many lifestyle and risk factor–modifying interventions that could protect the cerebrovascular endothelium and developing new specific drugs to preserve or enhance BBB function.

## References

[1] Lozano R., Naghavi M., Foreman K., Lim S., Shibuya K., Aboyans V. (2013). Global and regional mortality from 235 causes of death for 20 age groups in 1990 and 2010: a systematic analysis for the Global Burden of Disease Study 2010. Lancet.

[2] Iadecola C. (2013). The pathobiology of vascular dementia. Neuron.

[3] Makin S., Turpin S., Dennis M., Wardlaw J. (2013). Cognitive impairment after lacunar stroke: systematic review and meta-analysis of incidence, prevalence and comparison with other stroke sub-types. J Neurol Neurosurg Psychiatry.

[4] Valdes Hernandez M.C., Maconick L.C., Munoz Maniega S., Wang X., Wiseman S., Armitage P.A. (2015). A comparison of location of acute symptomatic versus ‘silent’ small vessel lesions. Int J Stroke.

[5] Debette S., Markus H.S. (2010). The clinical importance of white matter hyperintensities on brain magnetic resonance imaging: systematic review and meta-analysis. BMJ.

[6] Vermeer S.E., Longstreth W.T., Koudstaal P.J. (2007). Silent brain infarcts: a systematic review. Lancet Neurol.

[7] Cordonnier C., Al-Shahi Salman R., Wardlaw J. (2007). Spontaneous brain microbleeds: systematic review, subgroup analyses and standards for study design and reporting. Brain.

[8] Staals J., Booth T., Morris Z., Bastin M.E., Gow A.J., Corley J. (2015). Total MRI load of cerebral small vessel disease and cognitive ability in older people. Neurobiol Aging.

[9] Snowdon D.A., Greiner L.H., Mortimer J.A., Riley K.P., Greiner P.A., Markesbery W.R. (1997). Brain infarction and the clinical expression of Alzheimer disease. The Nun Study. JAMA.

[10] Matthews F.E., Brayne C., Lowe J., McKeith I., Wharton S.B., Ince P. (2009). Epidemiological pathology of dementia: attributable-risks at death in the Medical Research Council Cognitive Function and Ageing Study. PLoS Med.

[11] Smallwood A., Oulhaj A., Joachim C., Christie S., Sloan C., Smith A.D. (2012). Cerebral subcortical small vessel disease and its relation to cognition in elderly subjects: a pathological study in the Oxford Project to Investigate Memory and Ageing (OPTIMA) cohort. Neuropathol Appl Neurobiol.

[12] Tosto G., Zimmerman M.E., Carmichael O.T., Brickman A.M. (2014). Predicting aggressive decline in mild cognitive impairment: the importance of white matter hyperintensities. JAMA Neurol.

[13] Norton S., Matthews F.E., Barnes D.E., Yaffe K., Brayne C. (2014). Potential for primary prevention of Alzheimer's disease: an analysis of population-based data. Lancet Neurol.

[14] Ngandu T., Lehtisalo J., Solomon A., Levalahti E., Ahtiluoto S., Antikainen R. (2015). A 2 year multidomain intervention of diet, exercise, cognitive training, and vascular risk monitoring versus control to prevent cognitive decline in at-risk elderly people (FINGER): a randomised controlled trial. Lancet.

[15] Sposato L.A., Kapral M.K., Fang J., Gill S.S., Hackam D.G., Cipriano L.E. (2015). Declining incidence of stroke and dementia: coincidence or prevention opportunity?. JAMA Neurol.

[16] Pearce L.A., McClure L.A., Anderson D.C., Jacova C., Sharma M., Hart R.G. (2014). Effects of long-term blood pressure lowering and dual antiplatelet treatment on cognitive function in patients with recent lacunar stroke: a secondary analysis from the SPS3 randomised trial. Lancet Neurol.

[17] The SPS3 Investigators (2012). Effects of clopidogrel added to aspirin in patients with recent lacunar stroke. N Engl J Med.

[18] Thoonsen H., Richard E., Bentham P., Gray R., van Geloven N., De Haan R.J. (2010). Aspirin in Alzheimer's disease: increased risk of intracerebral hemorrhage: cause for concern?. Stroke.

[19] Wardlaw J.M., Smith C., Dichgans M. (2013). Mechanisms of sporadic cerebral small vessel disease: insights from neuroimaging. Lancet Neurol.

[20] Wharton S.B., Simpson J.E., Brayne C., Ince P.G. (2015). Age-associated white matter lesions: The MRC Cognitive Function and Ageing Study. Brain Pathol.

[21] Bath P.M., Wardlaw J.M. (2015). Pharmacological treatment and prevention of cerebral small vessel disease: a review of potential interventions. Int J Stroke.

[22] Wardlaw J.M., Sandercock P.A., Dennis M.S., Starr J. (2003). Is breakdown of the blood-brain barrier responsible for lacunar stroke, leukoaraiosis, and dementia?. Stroke.

[23] de la Torre J. (2010). The vascular hypothesis of Alzheimer's disease: bench to bedside and beyond. Neurodegener Dis.

[24] Bailey E.L., Smith C., Sudlow C.L., Wardlaw J.M. (2012). Pathology of lacunar ischaemic stroke in humans—a systematic review. Brain Pathol.

[25] Black S., Gao F., Bilbao J. (2009). Understanding white matter disease. Imaging-pathological correlations in vascular cognitive impairment. Stroke.

[26] Biron K.E., Dickstein D.L., Gopaul R., Jefferies W.A. (2011). Amyloid triggers extensive cerebral angiogenesis causing blood brain barrier permeability and hypervascularity in Alzheimer's disease. PLoS One.

[27] Kolstoe S.E., Ridha B.H., Bellotti V., Wang N., Robinson C.V., Crutch S.J. (2009). Molecular dissection of Alzheimer's disease neuropathology by depletion of serum amyloid P component. Proc Natl Acad Sci U S A.

[28] Farrall A.J., Wardlaw J.M. (2009). Blood brain barrier: ageing and microvascular disease—systemic review and meta-analysis. Neurobiol Aging.

[29] Starr J.M., Wardlaw J., Ferguson K., MacLullich A., Deary I.J., Marshall I. (2003). Increased blood-brain barrier permeability in type II diabetes demonstrated by gadolinium magnetic resonance imaging. J Neurol Neurosurg Psychiatry.

[30] Wardlaw J.M., Doubal F., Armitage P., Chappell F., Carpenter T., Maniega S.M. (2009). Lacunar stroke is associated with diffuse blood-brain barrier dysfunction. Ann Neurol.

[31] Topakian R., Barrick T.R., Howe F.A., Markus H.S. (2010). Blood-brain barrier permeability is increased in normal-appearing white matter in patients with lacunar stroke and leucoaraiosis. J Neurol Neurosurg Psychiatry.

[32] Taheri S., Gasparovic C., Huisa B.N., Adair J.C., Edmonds E., Prestopnik J. (2011). Blood-brain barrier permeability abnormalities in vascular cognitive impairment. Stroke.

[33] Kalaria R.N. (1999). The blood-brain barrier and cerebrovascular pathology in Alzheimer's disease. Ann N Y Acad Sci.

[34] Starr J.M., Farrall A.J., Armitage P., McGurn B., Wardlaw J. (2009). Blood-brain barrier permeability in Alzheimer's disease: a case-control MRI study. Psychiatry Res.

[35] Montagne A., Barnes S.R., Sweeney M.D., Halliday M.R., Sagare A.P., Zhao Z. (2015). Blood-brain barrier breakdown in the aging human hippocampus. Neuron.

[36] Heye A.K., Culling R.D., Valdes Hernandez M.C., Thrippleton M.J., Wardlaw J.M. (2014). Assessment of blood–brain barrier disruption using dynamic contrast-enhanced MRI. A systematic review. Neuroimage Clin.

[37] Heye A.K., Thrippleton M.J., Armitage P.A., Valdes Hernandez M.C., Makin S.D., Glatz A. (2016). Tracer kinetic modelling for DCE-MRI quantification of subtle blood-brain barrier permeability. Neuroimage.

[38] Maillard P., Seshadri S., Beiser A., Himali J.J., Au R., Fletcher E. (2012). Effects of systolic blood pressure on white-matter integrity in young adults in the Framingham Heart Study: a cross-sectional study. Lancet Neurol.

[39] Jackson C.A., Hutchison A., Dennis M.S., Wardlaw J.M., Lindgren A., Norrving B. (2010). Differing risk factor profiles of ischemic stroke subtypes: evidence for a distinct lacunar arteriopathy?. Stroke.

[40] Bamford J., Sandercock P., Dennis M., Burn J., Warlow C. (1991). Classification and natural history of clinically identifiable subtypes of cerebral infarction. Lancet.

[41] Bamford J., Sandercock P., Dennis M., Burn J., Warlow C. (1990). A prospective study of acute cerebrovascular disease in the community: the Oxfordshire Community Stroke Project—1981-86. 2. Incidence, case fatality rates and overall outcome at one year of cerebral infarction, primary intracerebral and subarachnoid haemorrhage. J Neurol Neurosurg Psychiatry.

[42] Valdés Hernández M., Armitage P., Thrippleton M.J., Chappell F., Sandeman E., Munoz Maniega S. (2015). Rationale, design and methodology of the image analysis protocol for studies of patients with cerebral small vessel disease and mild stroke. Brain Behav.

[43] Wardlaw J.M., Smith E.E., Biessels G.J., Cordonnier C., Fazekas F., Frayne R. (2013). Neuroimaging standards for research into small vessel disease and its contribution to ageing and neurodegeneration: a united approach. Lancet Neurol.

[44] Armitage P.A., Farrall A.J., Carpenter T.K., Doubal F.N., Wardlaw J.M. (2011). Use of dynamic contrast-enhanced MRI to measure subtle blood-brain barrier abnormalities. Magn Reson Imaging.

[45] Wardlaw J.M., Bastin M.E., Valdes Hernandez M.C., Munoz Maniega S., Royle N.A., Morris Z. (2011). Brain aging, cognition in youth and old age and vascular disease in the Lothian Birth Cohort 1936: rationale, design and methodology of the imaging protocol. Int J Stroke.

[46] Valdes Hernandez M.C., Ferguson K.J., Chappell F.M., Wardlaw J.M. (2010). New multispectral MRI data fusion technique for white matter lesion segmentation: method and comparison with thresholding in FLAIR images. Eur Radiol.

[47] Munoz Maniega S., Chappell F.M., Valdes Hernandez M.C., Armitage P.A., Makin S.D., Heye A.K. (2016). Integrity of normal-appearing white matter: influence of age, visible lesion burden and hypertension in patients with small vessel disease. J Cereb Blood Flow Metab.

[48] Munoz Maniega S., Valdes Hernandez M., Clayden J.D., Royle N.A., Murray C., Morris Z. (2015). White matter hyperintensities and normal-appearing white matter integrity in the aging brain. Neurobiol Aging.

[49] Barnes S.R., Ng T.S., Montagne A., Law M., Zlokovic B.V., Jacobs R.E. (2016). Optimal acquisition and modeling parameters for accurate assessment of low Ktrans blood-brain barrier permeability using dynamic contrast-enhanced MRI. Magn Reson Med.

[50] Heye A.K., Thrippleton M.J., Chappell F.M., Valdes Hernandez M.C., Armitage P.A., Makin S.D. (2016). Blood pressure and sodium: association with MRI markers in cerebral small vessel disease. J Cereb Blood Flow Metab.

[51] Benavente O.R., Coffey C.S., Conwit R., Hart R.G., McClure L.A., SPS3 Study Group (2013). Blood-pressure targets in patients with recent lacunar stroke: the SPS3 randomised trial. Lancet.

[52] Maillard P., Fletcher E., Lockhart S.N., Roach A.E., Reed B., Mungas D. (2014). White matter hyperintensities and their penumbra lie along a continuum of injury in the aging brain. Stroke.

[53] Huisa B.N., Caprihan A., Thompson J., Prestopnik J., Qualls C.R., Rosenberg G.A. (2015). Long-term blood-brain barrier permeability changes in Binswanger disease. Stroke.

[54] Lammie G.A., Brannan F., Wardlaw J.M. (1998). Incomplete lacunar infarction (type 1b lacunes). Acta Neuropathol.

[55] Chen A., Akinyemi R.O., Hase Y., Firbank M.J., Ndung'u M.N., Foster V. (2016). Frontal white matter hyperintensities, clasmatodendrosis and gliovascular abnormalities in ageing and post-stroke dementia. Brain.

[56] Turner S.T., Jack C.R., Fornage M., Mosley T.H., Boerwinkle E., de Andrade M. (2004). Heritability of leukoaraiosis in hypertensive sibships. Hypertension.

[57] Verhaaren B.F., Debette S., Bis J.C., Smith J.A., Ikram M.K., Adams H.H. (2015). Multi-ethnic genome-wide association study of cerebral white matter hyperintensities on MRI. Circ Cardiovasc Genet.

[58] Lopez L., Hill W.D., Harris S.E., Valdes Hernandez M., Munoz Maniega S., Bastin M.E. (2015). Genes from a translational analysis support a multifactorial nature of white matter hyperintensities. Stroke.

[59] Stevenson S.F., Doubal F.N., Shuler K., Wardlaw J.M. (2010). A systematic review of dynamic cerebral and peripheral endothelial function in lacunar stroke versus controls. Stroke.

