# Supplementary material for: Blood-brain barrier failure as a core mechanism in cerebral small vessel disease and dementia: evidence from a cohort study
Source: Alzheimers Dement. 2017 Jun;13(6):634–43. doi: 10.1016/j.jalz.2016.09.006 (PMC5472180; doi:10.1016/j.jalz.2016.09.006)
Supplement: Supplementary Data [file mmc1.doc]

**Title**: Blood-brain barrier failure as a core mechanism in cerebral small vessel disease and dementia: evidence from a cohort study.

**Online methods, tables and figures**

**Online Methods:** Image acquisition and analysis

**Online Table 1.** Magnetic resonance imaging sequence parameters.

**Online Table 2.** Demographic characteristics of the 201 patients with MRI data on blood-brain barrier leakage.

**Online Table 3.** Blood brain barrier leakage per brain tissue in 201 subjects. Estimates are the difference in change per minute in signal enhancement between the named tissue and normal appearing white matter (NAWM) determined by linear mixed modelling. Each estimate is adjusted for the named tissue and sagittal sinus signal, brain tissue T1 signal pre contrast (T_10_), age, Fazekas WMH score, stroke subtype, mean arterial pressure, pulse pressure, diagnosis of hypertension and smoking.. As the linear mixed modelling assumptions were not met for the index stroke lesion, the tissues were modelled separately to avoid contamination by the high leakage values in the index lesion.

**Online Table 4.** BBB leakage and ACE-R at 1-3 months after stroke

**Online Table 5.** BBB leakage at 1-3 months and ACE-R at 1 year after stroke

**Online Table 6.** BBB leakage at 1-3 months after stroke and decline in ACE-R from 1-3 months to one year after stroke

**Online Table 7.** Predictors of recurrent neurological outcomes and dependency at one year:

a) Logistic regression results for any recurrent clinical stroke, TIA, or new infarct detected on scanning;

b) Dependency, assessed by the modified Rankin Score ≥3.

**Online Figure 1.** Change in signal post-contrast in different brain tissues by time averaged across all patients. Left hand y-axis: NAWM, normal appearing white matter; WMH, white matter hyperintensities; DGM, deep grey matter; NSL, new (index) stroke lesion. Right-hand y-axis: SS, sagittal sinus, which represents the blood pool and provides an arterial input function for statistical modelling purposes. The graph demonstrates that signal in the blood pool declines following the first pass of bolus but increases progressively in all brain tissues meaning that intravascular contrast does not account for the tissue signal changes.

**Online Figure 2.** CONSORT Diagram recruitment and follow-up

**Online Figure 3.** BBB leakage (top left, Pby slope) , mean diffusivity (top right, MD), fractional anisotropy (bottom left, FA) and T1 (bottom right, ms) in WMH and in incremental 2-voxel wide contours (each contour approximately 2mm wide) from the WMH edge progressively distal into normal appearing white matter, adjusted for age.

**Online Methods: Clinical assessments, Image acquisition and analysis**

Clinical assessments

A specialist stroke physician recorded presenting symptoms, medical, family and drug history, smoking, alcohol use, pre-stroke functional status, performed the neurological examination and determined stroke severity (NIHSS). We assessed blood pressure in clinic, carotid stenosis (Doppler ultrasound), cardiac, blood haematology and biochemistry, urinary protein and eGFR.

The final stroke subtype (lacunar or cortical) was determined by a panel of stroke experts using the clinical syndrome[1] and acute infarct appearance on DWI MRI. In case of discrepancies, the imaging subtype was used.

We assessed cognitive status first at 1-3 months post-stroke. A trained clinician (SM) assessed the ACE-R.[2] We measured depression using the Beck Depression Index (BDI), and pre-morbid IQ using the (NART).[3] The NART has been validated in the 7^th^ and 8^th^ decades against actual cognitive ability at age 11 in a similar local population,[4] and is known to remain constant in early to moderate dementia, whilst other cognitive tests deteriorate. We were not able to visit patients in their homes, and therefore were only able to test patients who were able to return to hospital for cognitive testing. We defined cognitive impairment as an ACE-R score of <82, which has a sensitivity of 84% for dementia and a specificity of 100%.[2]

All patients were followed up one year after stroke to assess dependency (Oxford Handicap Scale, OHS,[5] like modified Rankin Scale[6]), recurrent stroke or TIA, other vascular events, vascular risk factors, blood pressure, we re-assessed the ACE-R. and obtained repeat MRI for WMH burden, new infarcts or haemorrhages. Patients not able to attend were followed up for clinical outcomes at home, by phone or postal questionnaire.

Image acquisition

All MR examinations were performed on a 1.5 Tesla GE Signa HDxt scanner, with self-shielding gradients (maximum strength 33 mT/m), and an 8-channel phased-array head coil, with tight quality assurance to maintain uniform performance. Diagnostic MRI at presentation with stroke included axial diffusion tensor imaging (DTI) to assess index stroke subtype and tissue integrity, Fluid Attenuated Inversion Recovery (FLAIR), T2-weighted, T2*-weighted, and sagittal T1-weighted imaging (Online Table 1) to assess previous infarct or haemorrhage, WMH, and other SVD features using standard criteria [7]. The protocol is published.[8]

Structural and DTI

The index infarct, old infarcts and SVD lesions were assessed using validated visual scores[9,10,7,11] by an expert neuroradiologist. We coded WMH using the Fazekas score.[10] Index small subcortical infarcts[7] were identified on DTI, as rounded, <2 cm diameter in basal ganglia, internal capsule, centrum semiovale or brainstem. Cortical infarcts had distribution of a cortical arterial territory or striatocapsular infarct (>2cm diameter).

We converted all images from DICOM to Analyze™7.5, registered structural sequences to the first pre-contrast FSPGR-12^o^ (<http://fsl.fmrib.ox.ac.uk/fsl/fslwiki/FLIRT>). We separated cerebrospinal fluid (CSF), whole brain, WMH, normal-appearing white and grey matter using a validated multispectral method,[12] ([www.sourceforge.net/projects/bric1936](http://www.sourceforge.net/projects/bric1936)) with manual editing as necessary (Figure 1), excluding the outermost voxels of CSF and deep grey matter to minimise cross-contamination. We segmented index and old infarcts manually. We differentiated WMH into ‘more’ and ‘less’ intense regions: ‘more’ intense (whiter) were visibly abnormal on FLAIR, T2- and T1- sequences against normal white matter; ‘less’ intense were visibly abnormal on FLAIR and isointense on T2- to normal-appearing white matter, and on T1- isointense to deep grey matter. We created 10 ‘contours’, each two voxels wide (2mm wide) from the WMH edge outwards through the normal-appearing white matter, for detailed spatial analysis of BBB leak and tissue integrity. We aligned the tissue maps to DTI space using non-linear registration, generated parametric mean diffusivity (MD) and fractional anisotropy (FA) maps.

BBB integrity

We extracted signal intensities from the DCE-MRI to assess BBB leakage per tissue, per voxel, per time after intravenous contrast.[13] The signal enhancement *E_t_* (fractional signal increase above baseline) was calculated as *E_t_=(S_t_-S*_0_*)/ S*_0_, for each time point t, where *S*_0_ is the signal intensity of the 12° pre-contrast acquisition, *S_t_* is the signal intensity per tissue for each post-contrast time point (Figure 2). The first four of the 21 post-contrast time points were omitted (as these reflect primarily intravascular contrast) leaving 17 timepoints representing the steady part of the curve (reflects slow BBB leakage dynamics). The long acquisition time helps detect subtle BBB leak.[14,15] We measured signal in sagittal sinus, CSF, deep grey matter, normal-appearing white matter, WMH, index and old infarcts. We used the individual patients’ sagittal sinus signal to correct for intravascular contrast[13] as it was better than the carotid arteries which provide a small target, variable signal, are motion-sensitive, suffer from in-flow and pulsation artefacts (especially in older subjects).[16] We calculated pre-contrast T1 (longitudinal relaxation time, T_10_,[13] milliseconds) for each patient using the variable flip angle method [17] to control for tissue characteristics that influence post-contrast signal enhancement.[14,13]

Statistical analysis of BBB leak

Of several models that estimate BBB permeability,[14,18] the Patlak best suits low permeability states,[14,19,20] but all models rely on assumptions that are become invalid in low permeability states, e.g. that capillary density, which relates to surface area and blood volume, are constant, whereas both vary between tissues, decrease with age and in disease.[16] Strong effects of age on BBB, T1, MD and FA were shown in two separate analyses.[21,22] Therefore, in our pre-specified analysis (protocol online) we did not calculate permeability but used linear mixed modelling of the signal enhancement slopes (Figure 2) to identify tissue-specific and patient-specific differences in BBB leakage between pre-specified groups. All analyses were adjusted for age, WMH burden, vascular risk factors, intravascular contrast, baseline tissue T1 and time after contrast injection. This approach accounted for each patient contributing data from 17 time-points per tissue, each representing 73 seconds (which we adjusted to signal change per minute).

We fitted separate models for each brain tissue, as the models including all tissues simultaneously had poor residual distribution due to marked differences in BBB leak in some tissues. The main predictors were age at presentation, stroke subtype, and WMH burden (Fazekas periventricular and deep white matter score summed), adjusted for each other and for: mean arterial pressure at presentation (2*diastolic +systolic)/3); smoking (never/ex-smoker versus current/ex-smoker for <1 year); pre-contrast tissue T1; and sagittal sinus signal to adjust for vascular input function. Separate analyses were performed for each interaction term as the data were already adjusted for key predictors and simultaneous fitting of multiple interaction terms was not supported. We calculated 95% confidence intervals (95%CI) of medians using the Hodges-Lehmann estimate, and p-values using the Wilcoxon rank sum test (i.e. Mann-Whitney U test). We calculated 95%CI of proportions using a simple difference in proportions test and p-value using a simple chi-square test. We used SAS 9.3 ([www.sas.com](http://www.sas.com/)) for all analyses and R 2.13.1 for graphs.

Sample size

We calculated sample size using Power3 (http://dceg.cancer.gov/tools/design/power) for studying features of relative odds of disease using multivariate modelling, assuming an OR for composite disease progression in lacunar vs. cortical stroke of 12.25 (based on 10-15% of patients having clinical and/or imaging evidence of small vessel disease progression at 1 year as a composite outcome), Type I and II errors rates of 0.05 and 0.8 respectively. For 80% power, a two-sided test, the estimated sample size is 170 patients to reach one year follow-up. Allowing for about 10% drop-out required 200 patients to have BBB imaging.

**Online Table 1.** Magnetic resonance imaging sequence parameters.

| **Sequence** | **T1W spin echo** | **DWI/DTI**  **(30 diffusion directions)** | **FLAIR (TI=2200 ms)** | **T2W Fast spin echo** | **T2*W gradient recalled echo (FA=20^o^)** | **3D IR PREP (TI=500ms, FA=8 ^o^)** | **SPGR**  **(FA=2 ^o^)** | **SPGR* (FA=12 ^o^)** |
| --- | --- | --- | --- | --- | --- | --- | --- | --- |
| Orientation | Sagittal | Axial | Axial | Axial | Axial | Sagittal | Axial | Axial |
| TE (ms) | 14 | 82 | 153 | 90 | 15 | 2.9 | 3.1 | 3.1 |
| TR (ms) | 400 | 7700 | 9000 | 6000 | 800 | 7.3 | 8.2 | 8.2 |
| FOV | 24 x 24 | 24 x 24 | 24 x 24 | 24 x 24 | 24(AP) x 18 | 330(SI) x 214.5 | 24 x 24 | 24 x 24 |
| Slice thickness (mm) | 5.0 | 5.0 | 5.0 | 5.0 | 5.0 | 1.8 | 4 | 4 |
| Slice gap (mm) | 0 | 1.0 | 1.0 | 1.0 | 1.0 | 0 | 0 | 0 |
| Matrix | 256(SI) x 160 | 128x128 | 384x224 | 384X384 | 384(AP)x168 | 256(SI)x146 | 256(AP)X192 | 256(AP)X192 |
|  |  |  |  |  |  |  |  |  |
| No. slices | 5 | 28 | 28 | 28 | 28 | 100 | 42 | 42 |
| Acquisition time | 0:54 | 4:14 | 4:48 | 2:30 | 4:32 | 4:17 | 1.13 | 1.13 |

^+^ sequence repeated after contrast injection 20 times consecutively.

**Online Table 2.** Demographic characteristics of the 201 patients with MRI data on blood-brain barrier leakage.

|  | **All** | **Lacunar** | **Cortical** | **Difference (95% CI)** | **P value** |
| --- | --- | --- | --- | --- | --- |
| N | 201 | 92 | 109 |  |  |
| Age (IQR) | 66.8 (56.8-75.2) | 64.0 (55.7-72.2) | 67.5 (59.6-76.0) | -2.99 (-6.27 to 0.45) | 0.097 |
| Male | 122 (60.7%) | 54 (58.70%) | 68 (62.4%) | -3.69% (-17.25% to 9.87%) | 0.59 |
| Female | 79 (39.3%) | 38 (41.3%) | 41 (37.6%) | 3.69% (-9.87% to 17.25%) | 0.59 |
| NIHSS worst median (IQR) | 2 (1-3) | 2 (2-4) | 2 (1-3) | 0.0 (0.0 to 1.0) | 0.025 |
| NIHSS at assessment median (IQR) | 1 (0-1) | 1 (0-2) | 1 (0-1) | 0.0 (0.0 to 1.0) | 0.0018 |
| mRS at presentation median (IQR) | 1 (1-2) | 1 (1-2) | 1 (1-2) | 0.0 (0.0 to 0.0) | 0.85 |
| Time (days) stroke to assessment | 3 (1-7) | 3 (1-6) | 3 (1-8) | 0.0 (-1.0 to 1.0) | 0.71 |
| Hypertension | 150 (73.1%) | 68 (73.9%) | 82 (75.2%) | -1.3% (-13.4% to 10.8%) | 0.83 |
| Diabetes | 25 (12.4%) | 10 (10.9%) | 15 (13.8%) | -2.9% (-12.0% to 6.2%) | 0.54 |
| Hyperlipidaemia | 121 (60.2%) | 60 (65.2%) | 61 (56.0%) | 9.3% (-4.2 to 22.7%) | 0.18 |
| Smoker (current or stopped in last year) | 72 (35.8%) | 38 (41.3%) | 34 (31.2%) | 10.1% (-3.19 to 23.4%) | 0.14 |
| Atrial fibrillation | 18 (9.0%) | 5 (5.4%) | 13 (11.9%) | -6.5% (-14.1% to 1.2%) | 0.11 |
| Ischaemic heart disease | 44 (21.9%) | 17 (18.5%) | 27 (24.8%) | -6.3% (-17.6% to 5.1%) | 0.28 |
| Left ventricular systolic dysfunction | 7 (3.5%) | 5 (5.4%) | 2 (1.8%) | 3.6% (-1.7% to 8.9%) | 0.17 |
| Peripheral vascular disease | 12 (6.0%) | 3 (3.3%) | 9 (8.3%) | -5.0% (-11.3% to 1.3%) | 0.14 |
| Carotid stenosis >50% ipsilateral | 17 (0.08%) | 6 (6.9%) | 14 (15.0%) | -6.32% (-14.4 to -1.7%) | 0.01 |
| Any ipsilateral embolic source | 39 (19.4%) | 11 (12.0%) | 28 (25.7%) | -13.7% (-24.3 to -3.2%) | 0.014 |
| Prior TIA | 23 (11.4%) | 10 (10.9%) | 13 (11.9%) | -1.1% (-9.9% to 7.7%) | 0.81 |
| Prior stroke | 27 (13.4%) | 15 (16.3%) | 12 (11.0%) | 5.3% (-4.3% to 14.9%) | 0.27 |
| Systolic blood pressure median (IQR) | 141 (130-159) | 145 (130-158.5) | 136 (124-159) | 5.0 (-2.0 to 11.0) | 0.19 |
| Diastolic blood pressure median (IQR) | 80 (70-90) | 82.5 (70.5-94) | 80 (70-90) | 3.0 (0.0 to 8.0) | 0.12 |
| Mean arterial pressure median (IQR) | 100 (91.3 to 113) | 104.8 (91.5-115.8) | 97 (91.3 to 112) | 3.3 (-1.0 to 8.0) | 0.16 |
| Pulse pressure median (IQR) | 60 (49 to 76) | 60 (49 to 75.5) | 60 (48 to 76) | 0.0 (-5.0 to 6.0) | 0.96 |

Numbers are n (%) unless stated otherwise. We calculated 95% confidence intervals (CI) of medians using the Hodges-Lehmann estimate, and p-value using the Wilcoxon rank sum test (ie Mann-Whitney U test). We calculated 95% CI of proportions using a simple difference in proportions test and the p-value using a simple chi-square test. The percentages refer to the proportion of all patients, lacunar patients, and cortical patients with a given characteristic. IQR = interquartile range, NIHSS = National Institutes of Health Stroke Scale, mRS = modified Rankin Scale, TIA = transient ischaemic attack.

**Online Table 3.** Blood brain barrier leakage per brain tissue in 201 subjects.

Estimates are the difference in change per minute in signal enhancement between the named tissue and normal appearing white matter (NAWM) determined by linear mixed modelling. Each estimate is adjusted for the named tissue and sagittal sinus signal, brain tissue T1 signal pre contrast (T_10_), age, Fazekas WMH score, stroke subtype, mean arterial pressure, pulse pressure, diagnosis of hypertension and smoking.. As the linear mixed modelling assumptions were not met for the index stroke lesion, the tissues were modelled separately to avoid contamination by the high leakage values in the index lesion.

| **Predictor** | **β co-efficient**  **x 10^3^** | **95% CI x 1000** | **P value** |
| --- | --- | --- | --- |
| Change in signal enhancement per minute in NAWM adjusted for **deep grey matter** and other predictors | 0.385 | (0.292,0.478) | <0.0001 |
| Difference in signal enhancement: deep grey matter minus NAWM at time=0 | 37.13 | (36.29,37.97) | <0.0001 |
| Change per minute in difference (deep grey matter minus NAWM) in signal enhancement | -0.17 | (-0.24,-0.09) | <0.0001 |
| Change in signal enhancement per minute in NAWM adjusted for **CSF** and other predictors | 0.489 | (0.192,0.787) | 0.0013 |
| Difference in signal enhancement: CSF minus NAWM at time=0 | 203.5 | (200.7,206.3) | <0.0001 |
| Change per minute in difference (CSF minus NAWM) in signal enhancement | 0.823 | (0.577,1.068) | <0.0001 |
| Change in signal enhancement per minute in NAWM adjusted for **WMH** and other predictors | 0.351 | (0.251,0.451) | <0.0001 |
| Difference in signal enhancement: WMH minus NAWM at time=0 | 13.46 | (12.56,14.36) | <0.0001 |
| Change per minute in difference (WMH minus NAWM) in signal enhancement | 0.09 | (0.011,0.169) | 0.025 |
| Change in signal enhancement per minute in NAWM adjusted for **index stroke lesion** and other predictors | 0.111 | (-0.09,0.313) | 0.28 |
| Difference in signal enhancement: index stroke lesion minus NAWM at time=0 | 29.17 | (27.11,31.24) | <0.0001 |
| Change per minute in difference (index stroke lesion minus NAWM) in signal enhancement | 0.631 | (0.454,0.808) | <0.0001 |
| Change in signal enhancement per minute in NAWM adjusted for **old stroke lesion** and other predictors | 0.283 | (0.155,0.411) | <0.0001 |
| Difference in signal enhancement: old stroke lesion minus NAWM at time=0 | 19.63 | (18.1,21.17) | <0.0001 |
| Change per minute in difference (old stroke lesion minus NAWM) in signal enhancement | 0.247 | (0.12,0.375) | 0.0001 |

**Online Table 4.** BBB leakage per tissue type and ACE-R at 1-3 months after stroke

| **Tissue** | **Variable** | **Beta Coefficient (95%CI) x 10^3^** | **P** |
| --- | --- | --- | --- |
| Deep Grey Matter | BBB Leakage in Grey Matter | -0.17(-2.85,2.52) | 0.9 |
|  | Cortical sub-type | 0.01(-2.27,2.29) | 0.99 |
|  | Age | -0.19(-0.3,-0.07) | ***<0.001*** |
|  | Units of alcohol | 0.02(-0.08,0.13) | 0.68 |
|  | Fazekas score at index stroke | -0.64(-1.43,0.16) | 0.11 |
|  | Hypertension | 1.11(-1.41,3.62) | 0.39 |
|  | NART at 1-3 months | 0.34(0.22,0.46) | ***<0.001*** |
|  | BDI at 1-3 months | -0.07(-0.19,0.05) | 0.25 |
|  | Leakage x subtype | 1.49(-1.95,4.93) | 0.39 |
| Normal appearing white matter | BBB Leakage in NAWM | -0.29(-3.69,3.12) | 0.87 |
|  | Cortical sub-type | -0.27(-2.85,2.32) | 0.84 |
|  | Age | -0.19(-0.3,-0.07) | ***0.0015*** |
|  | Units of alcohol | 0.03(-0.08,0.13) | 0.59 |
|  | Fazekas score at index stroke | -0.61(-1.4,0.17) | 0.12 |
|  | Hypertension | 1.26(-1.22,3.75) | 0.32 |
|  | NART at 1-3 months | 0.34(0.22,0.46) | ***<0.001*** |
|  | BDI at 1-3 months | -0.07(-0.19,0.05) | 0.26 |
|  | Leakage x subtype | 1.46(-3.15,6.07) | 0.53 |
| CSF | BBB Leakage in CSF | -0.15(-0.76,0.46) | 0.63 |
|  | Cortical sub-type | -0.19(-2.75,2.36) | 0.88 |
|  | Age | -0.19(-0.3,-0.07) | ***<0.001*** |
|  | Units of alcohol | 0.03(-0.08,0.13) | 0.6 |
|  | Fazekas score at index stroke | -0.58(-1.37,0.2) | 0.14 |
|  | Hypertension | 1.36(-1.1,3.82) | 0.28 |
|  | NART at 1-3 months | 0.34(0.22,0.46) | ***<0.001*** |
|  | BDI at 1-3 months | -0.07(-0.19,0.05) | 0.27 |
|  | Leakage x subtype | 0.25(-0.54,1.04) | 0.54 |
| White matter hyperintensities | BBB Leakage in WMH | -1.54(-4.04,0.96) | 0.23 |
|  | Cortical sub-type | -0.59(-3.11,1.93) | 0.64 |
|  | Age | -0.18(-0.3,-0.07) | ***<0.001*** |
|  | Units of alcohol | 0.03(-0.07,0.13) | 0.54 |
|  | Fazekas score at index stroke | -0.56(-1.36,0.23) | 0.16 |
|  | Hypertension | 1.36(-1.12,3.85) | 0.28 |
|  | NART at 1-3 months | 0.34(0.22,0.45) | ***<0.001*** |
|  | BDI at 1-3 months | -0.08(-0.2,0.04) | 0.2 |
|  | Leakage x subtype | 2.17(-1.05,5.38) | 0.18 |
| Index stroke lesion | BBB Leakage in the new stroke lesion | -0.35(-2.36,1.65) | 0.73 |
|  | Cortical sub-type | 0.3(-2.31,2.91) | 0.82 |
|  | Age | -0.19(-0.3,-0.07) | ***0.0013*** |
|  | Units of alcohol | 0.03(-0.07,0.13) | 0.58 |
|  | Fazekas score at index stroke | -0.56(-1.35,0.22) | 0.16 |
|  | Hypertension | 1.61(-0.93,4.16) | 0.21 |
|  | NART at 1-3 months | 0.34(0.22,0.45) | ***<0.001*** |
|  | BDI at 1-3 months | -0.07(-0.19,0.05) | 0.26 |
|  | Leakage x subtype | 0.04(-2.13,2.22) | 0.97 |
| Old stroke lesion | BBB Leakage in the old stroke lesion | 2.56(-1.18,6.3) | 0.18 |
|  | Cortical sub-type | 0.44(-1.92,2.8) | 0.71 |
|  | Age | -0.18(-0.3,-0.07) | ***<0.001*** |
|  | Units of alcohol | 0.02(-0.08,0.12) | 0.7 |
|  | Fazekas score at index stroke | -0.71(-1.51,0.09) | 0.08 |
|  | Hypertension | 1.19(-1.28,3.66) | 0.34 |
|  | NART at 1-3 months | 0.34(0.23,0.46) | ***<0.001*** |
|  | BDI at 1-3 months | -0.07(-0.19,0.06) | 0.28 |
|  | Leakage x subtype | -2.25(-6.26,1.75) | 0.27 |

**Online Table 5.** BBB leakage per tissue type at 1-3 months and ACE-R at 1 year after stroke

| **Tissue** | **Variable** | **Beta Coefficient (95%CI) x 10^3^** | **P** |
| --- | --- | --- | --- |
| Grey Matter | BBB Leakage | -2.1 (95%CI ,-4.72 , 0.52) | 0.11 |
|  | Cortical sub-type | -1.01 (95%CI ,-3.3 , 1.28) | 0.38 |
|  | Age | -0.1 (95%CI ,-0.22 , 0.01) | 0.07 |
|  | Units of alcohol | 0.07 (95%CI ,-0.02 , 0.16) | 0.12 |
|  | Fazekas score at index stroke | -0.86 (95%CI ,-1.63 , -0.09) | ***0.03*** |
|  | Hypertension | 1.6 (95%CI ,-0.94 , 4.15) | 0.21 |
|  | NART at 1-3 months | 0.3 (95%CI ,0.18 , 0.41) | ***<0.001*** |
|  | BDI at 1-3 months | -0.06 (95%CI ,-0.19 , 0.07) | 0.35 |
|  | Leakage x subtype | 2.92 (95%CI ,-0.49 , 6.33) | 0.09 |
| Normal appearing white matter | BBB Leakage in NAWM | -1.69 (95%CI ,-5.05 , 1.67) | 0.32 |
|  | Cortical sub-type | -1.37 (95%CI ,-4.02 , 1.28) | 0.31 |
|  | Age | -0.1 (95%CI ,-0.21 , 0.01) | 0.09 |
|  | Units of alcohol | 0.06 (95%CI ,-0.03 , 0.15) | 0.17 |
|  | Fazekas score at index stroke | -0.89 (95%CI ,-1.67 , -0.11) | ***0.03*** |
|  | Hypertension | 1.68 (95%CI ,-0.89 , 4.25) | 0.2 |
|  | NART at 1-3 months | 0.31 (95%CI ,0.2 , 0.42) | ***<0.001*** |
|  | BDI at 1-3 months | -0.07 (95%CI ,-0.2 , 0.06) | 0.3 |
|  | Leakage x subtype | 2.11 (95%CI ,-2.35 , 6.57) | 0.35 |
| CSF | BBB Leakage | -0.45 (95%CI ,-1.03 , 0.12) | 0.12 |
|  | Cortical sub-type | -1.87 (95%CI ,-4.41 , 0.67) | 0.15 |
|  | Age | -0.11 (95%CI ,-0.22 , 0.01) | 0.07 |
|  | Units of alcohol | 0.05 (95%CI ,-0.03 , 0.14) | 0.21 |
|  | Fazekas score at index stroke | -0.83 (95%CI ,-1.6 , -0.07) | ***0.03*** |
|  | Hypertension | 1.51 (95%CI ,-0.95 , 3.97) | 0.23 |
|  | NART at 1-3 months | 0.3 (95%CI ,0.19 , 0.42) | ***<0.001*** |
|  | BDI at 1-3 months | -0.07 (95%CI ,-0.19 , 0.06) | 0.29 |
|  | Leakage x subtype | 0.71 (95%CI ,-0.08 , 1.5) | 0.08 |
| White matter hyperintensities | BBB Leakage | -3.52 (95%CI ,-5.9 , -1.13) | ***<0.001*** |
|  | Cortical sub-type | -1.99 (95%CI ,-4.49 , 0.51) | 0.12 |
|  | Age | -0.08 (95%CI ,-0.19 , 0.03) | 0.14 |
|  | Units of alcohol | 0.07 (95%CI ,-0.01 , 0.15) | 0.1 |
|  | Fazekas score at index stroke | -0.78 (95%CI ,-1.54 , -0.01) | ***0.05*** |
|  | Hypertension | 2.05 (95%CI ,-0.43 , 4.53) | 0.1 |
|  | NART at 1-3 months | 0.29 (95%CI ,0.18 , 0.4) | ***<0.001*** |
|  | BDI at 1-3 months | -0.07 (95%CI ,-0.2 , 0.05) | 0.25 |
|  | Leakage x subtype | 3.19 (95%CI ,0.13 , 6.25) | ***0.04*** |
| Index Stroke Lesion | BBB Leakage | -0.14 (95%CI ,-2.3 , 2.01) | 0.9 |
|  | Cortical sub-type | -0.29 (95%CI ,-3.07 , 2.49) | 0.84 |
|  | Age | -0.1 (95%CI ,-0.21 , 0.01) | 0.07 |
|  | Units of alcohol | 0.06 (95%CI ,-0.03 , 0.14) | 0.22 |
|  | Fazekas score at index stroke | -0.81 (95%CI ,-1.59 , -0.04) | ***0.04*** |
|  | Hypertension | 1.94 (95%CI ,-0.65 , 4.53) | 0.14 |
|  | NART at 1-3 months | 0.3 (95%CI ,0.18 , 0.42) | ***<0.001*** |
|  | BDI at 1-3 months | -0.08 (95%CI ,-0.21 , 0.05) | 0.24 |
|  | Leakage x subtype | -0.38 (95%CI ,-2.68 , 1.92) | 0.75 |
| Old Stroke Lesion | BBB Leakage | 6.44 (95%CI ,2.45 , 10.43) | ***<0.001*** |
|  | Cortical sub-type | -0.61 (95%CI ,-2.87 , 1.64) | 0.59 |
|  | Age | -0.1 (95%CI ,-0.21 , 0.01) | 0.07 |
|  | Units of alcohol | 0.05 (95%CI ,-0.04 , 0.13) | 0.26 |
|  | Fazekas score at index stroke | -1.11 (95%CI ,-1.87 , -0.35) | ***<0.001*** |
|  | Hypertension | 1.16 (95%CI ,-1.23 , 3.56) | 0.34 |
|  | NART at 1-3 months | 0.33 (95%CI ,0.22 , 0.44) | ***<0.001*** |
|  | BDI at 1-3 months | -0.05 (95%CI ,-0.17 , 0.07) | 0.42 |
|  | Leakage x subtype | -5.49 (95%CI ,-9.72 , -1.27) | ***0.01*** |

**Online Table 6.** BBB leakage per tissue type at 1-3 months after stroke and decline in ACE-R from 1-3 months to one year after stroke

| **Tissue** | **Variable** | **Beta coefficient (95%CI) x 10^3^** | **p** |
| --- | --- | --- | --- |
| Grey Matter | BBB Leakage | -0.77 (95%CI-3.11,1.58) | 0.52 |
|  | Cortical Sub-type | -0.76 (95%CI-2.67,1.14) | 0.43 |
|  | Age | 0.01 (95%CI-0.07,0.1) | 0.73 |
|  | Units | 0.04 (95%CI-0.03,0.11) | 0.27 |
|  | Total Fazekas Score | -0.47 (95%CI-1.03,0.08) | 0.09 |
|  | Hypertension | 1.24 (95%CI-0.6,3.09) | 0.18 |
|  | NART at 1-3 months | 0.09 (95%CI0.01,0.18) | ***0.04*** |
|  | BDI at 1-3 months | -0.06 (95%CI-0.15,0.03) | 0.21 |
|  | ACE-R at 1-3 months | -0.06 (95%CI-0.15,0.85) | ***<0.001*** |
|  | Leakage x subtype | -0.06 (95%CI-0.15,3.73) | 0.27 |
| Normal appearing white matter | BBB Leakage | -0.77 (95%CI-3.11,1.58) | 0.52 |
|  | Cortical Sub-type | -0.76 (95%CI-2.67,1.14) | 0.43 |
|  | Age | 0.01 (95%CI-0.07,0.1) | 0.73 |
|  | Units | 0.04 (95%CI-0.03,0.11) | 0.27 |
|  | Total Fazekas Score | -0.47 (95%CI-1.03,0.08) | 0.09 |
|  | Hypertension | 1.24 (95%CI-0.6,3.09) | 0.18 |
|  | NART at 1-3 months | 0.09 (95%CI0.01,0.18) | ***0.04*** |
|  | BDI at 1-3 months | -0.06 (95%CI-0.15,0.03) | 0.21 |
|  | ACE-R at 1-3 months | -0.06 (95%CI-0.15,0.85) | ***<0.001*** |
|  | Leakage x subtype | -0.06 (95%CI-0.15,3.62) | 0.77 |
| CSF | BBB Leakage | -0.27 (95%CI-0.68,0.13) | 0.18 |
|  | Cortical sub-type | -1.45 (95%CI-3.24,0.35) | 0.11 |
|  | Age | 0.01 (95%CI-0.08,0.09) | 0.85 |
|  | Units | 0.03 (95%CI-0.04,0.1) | 0.35 |
|  | Total Fazekas Score | -0.46 (95%CI-1,0.08) | 0.09 |
|  | Hypertension | 1.01 (95%CI-0.74,2.77) | 0.26 |
|  | NART at 1-3 months | 0.09 (95%CI0,0.18) | ***0.04*** |
|  | BDI at 1-3 months | -0.06 (95%CI-0.15,0.03) | 0.2 |
|  | ACE-R at 1-3 months | -0.06 (95%CI-0.15,0.84) | ***<0.001*** |
|  | Leakage x subtype | -0.06 (95%CI-0.15,1.11) | 0.05 |
| White matter hyperintensities | BBB Leakage | -1.89 (95%CI-3.62,-0.16) | ***0.03*** |
|  | Cortical sub-type | -1.09 (95%CI-2.92,0.74) | 0.24 |
|  | Age | 0.02 (95%CI-0.06,0.1) | 0.57 |
|  | Units | 0.05 (95%CI-0.02,0.12) | 0.16 |
|  | Total Fazekas Score | -0.39 (95%CI-0.94,0.16) | 0.16 |
|  | Hypertension | 1.53 (95%CI-0.26,3.32) | 0.09 |
|  | NART at 1-3 months | 0.09 (95%CI0,0.17) | ***0.05*** |
|  | BDI at 1-3 months | -0.06 (95%CI-0.15,0.03) | 0.19 |
|  | ACE-R at 1-3 months | -0.06 (95%CI-0.15,0.83) | ***<0.001*** |
|  | Leakage x subtype | -0.06 (95%CI-0.15,3.23) | 0.38 |
| Index Stroke Lesion | BBB Leakage | 0.47 (95%CI-1.06,1.99) | 0.55 |
|  | Cortical Sub-type | -0.19 (95%CI-2.15,1.77) | 0.85 |
|  | Age | 0.01 (95%CI-0.07,0.1) | 0.74 |
|  | Units | 0.03 (95%CI-0.04,0.1) | 0.38 |
|  | Total Fazekas Score | -0.45 (95%CI-1,0.1) | 0.11 |
|  | Hypertension | 1.1 (95%CI-0.76,2.97) | 0.24 |
|  | NART at 1-3 months | 0.09 (95%CI0,0.18) | ***0.04*** |
|  | BDI at 1-3 months | -0.06 (95%CI-0.15,0.03) | 0.18 |
|  | ACE-R at 1-3 months | -0.06 (95%CI-0.15,0.85) | ***<0.001*** |
|  | Leakage x subtype | -0.06 (95%CI-0.15,0.95) | 0.41 |
| Old Stroke Lesion | BBB Leakage | 3.88 (95%CI1.04,6.73) | ***0.01*** |
|  | Cortical Sub-type | -0.51 (95%CI-2.14,1.11) | 0.53 |
|  | Age | 0.01 (95%CI-0.07,0.09) | 0.76 |
|  | Units | 0.03 (95%CI-0.04,0.09) | 0.46 |
|  | Total Fazekas Score | -0.65 (95%CI-1.2,-0.1) | ***0.02*** |
|  | Hypertension | 0.84 (95%CI-0.89,2.57) | 0.34 |
|  | NART at 1-3 months | 0.11 (95%CI0.03,0.2) | ***0.01*** |
|  | BDI at 1-3 months | -0.04 (95%CI-0.13,0.04) | 0.31 |
|  | ACE-R at 1-3 months | -0.04 (95%CI-0.13,0.81) | ***<0.001*** |
|  | Leakage x subtype | -0.04 (95%CI-0.13,-0.31) | ***0.03*** |

**Online Table 7.** Predictors of neurological outcomes at one year:

a) Logistic regression results with binary neuro-outcome (any recurrent clinical stroke, TIA, or new infarct detected on scanning);

| **Tissue** | **Predictor** | **β co-efficient** | **Lower 95%CI** | **Upper 95% CI** | **P value** |
| --- | --- | --- | --- | --- | --- |
| Deep grey matter | Age (unit = 5 years) | 1.091 | 0.863 | 1.38 | 0.47 |
|  | Fazekas WMH score | 1.32 | 1.01 | 1.725 | 0.042 |
|  | Mean arterial pressure | 0.99 | 0.961 | 1.02 | 0.50 |
|  | Hypertension diagnosis | 1.616 | 0.578 | 4.513 | 0.36 |
|  | Pulse pressure | 0.997 | 0.974 | 1.02 | 0.79 |
|  | Lacunar v cortical | 1.193 | 0.538 | 2.646 | 0.66 |
|  | Smoker | 1.25 | 0.506 | 3.086 | 0.63 |
|  | BBB leak. slope estimate | 0.831 | 0.426 | 1.621 | 0.59 |
| Normal appearing white matter | Age (unit = 5 years) | 1.092 | 0.861 | 1.384 | 0.47 |
|  | Fazekas WMH score | 1.323 | 1.011 | 1.73 | 0.041 |
|  | Mean arterial pressure | 0.989 | 0.961 | 1.019 | 0.47 |
|  | Hypertension diagnosis | 1.802 | 0.632 | 5.143 | 0.27 |
|  | Pulse pressure | 0.996 | 0.974 | 1.019 | 0.74 |
|  | Lacunar v cortical | 1.149 | 0.515 | 2.563 | 0.73 |
|  | Smoker | 1.239 | 0.499 | 3.073 | 0.64 |
|  | BBB leak. slope estimate | 0.602 | 0.263 | 1.379 | 0.23 |
| White matter hyperintensities | Age (unit = 5 years) | 1.092 | 0.865 | 1.379 | 0.46 |
|  | Fazekas WMH score | 1.321 | 1.004 | 1.738 | 0.047 |
|  | Mean arterial pressure | 0.99 | 0.961 | 1.02 | 0.50 |
|  | Hypertension diagnosis | 1.563 | 0.563 | 4.339 | 0.39 |
|  | Pulse pressure | 0.996 | 0.974 | 1.019 | 0.76 |
|  | Lacunar v cortical | 1.209 | 0.547 | 2.67 | 0.64 |
|  | Smoker | 1.218 | 0.494 | 3.001 | 0.67 |
|  | BBB leak. slope estimate | 0.881 | 0.46 | 1.685 | 0.70 |
| CSF | Age (unit = 5 years) | 1.083 | 0.858 | 1.368 | 0.50 |
|  | Fazekas WMH score | 1.307 | 1.004 | 1.701 | 0.047 |
|  | Mean arterial pressure | 0.99 | 0.961 | 1.019 | 0.49 |
|  | Hypertension diagnosis | 1.559 | 0.566 | 4.296 | 0.39 |
|  | Pulse pressure | 0.995 | 0.973 | 1.018 | 0.69 |
|  | Lacunar v cortical | 1.195 | 0.54 | 2.645 | 0.66 |
|  | Smoker | 1.247 | 0.506 | 3.07 | 0.63 |
|  | BBB leak. slope estimate | 1.055 | 0.899 | 1.237 | 0.51 |
| Index stroke lesion | Age (unit = 5 years) | 1.029 | 0.797 | 1.327 | 0.83 |
|  | Fazekas WMH score | 1.303 | 0.964 | 1.761 | 0.085 |
|  | Mean arterial pressure | 0.994 | 0.961 | 1.029 | 0.75 |
|  | Hypertension diagnosis | 0.96 | 0.303 | 3.043 | 0.94 |
|  | Pulse pressure | 0.99 | 0.965 | 1.016 | 0.46 |
|  | Lacunar v cortical | 0.998 | 0.392 | 2.541 | 0.996 |
|  | Smoker | 1.085 | 0.39 | 3.015 | 0.88 |
|  | BBB leak. slope estimate | 0.931 | 0.649 | 1.336 | 0.70 |
| Old (pre-existing) stroke lesion | Age (unit = 5 years) | 1.323 | 0.912 | 1.917 | 0.14 |
|  | Fazekas WMH score | 1.055 | 0.719 | 1.549 | 0.78 |
|  | Mean arterial pressure | 0.957 | 0.911 | 1.005 | 0.078 |
|  | Hypertension diagnosis | 1.583 | 0.374 | 6.703 | 0.53 |
|  | Pulse pressure | 1.006 | 0.972 | 1.04 | 0.74 |
|  | Lacunar v cortical | 1.969 | 0.527 | 7.353 | 0.31 |
|  | Smoker | 0.966 | 0.239 | 3.894 | 0.96 |
|  | BBB leak. slope estimate | 1.153 | 0.6 | 2.218 | 0.67 |

b) Dependency, assessed by the modified Rankin Score ≥3.

| **Tissue** | **Predictor** | **β co-efficient** | **Lower 95%CI** | **Upper 95% CI** | **P value** |
| --- | --- | --- | --- | --- | --- |
| Deep grey matter | Age (unit = 5 years) | 1.062 | 0.92 | 1.225 | 0.41 |
|  | Fazekas WMH score | 1.229 | 1.029 | 1.468 | 0.023 |
|  | Mean arterial pressure | 0.987 | 0.968 | 1.006 | 0.18 |
|  | Hypertension diagnosis | 1.36 | 0.735 | 2.517 | 0.33 |
|  | Pulse pressure | 0.991 | 0.977 | 1.006 | 0.25 |
|  | Lacunar v cortical | 0.894 | 0.531 | 1.506 | 0.67 |
|  | Smoker | 1.61 | 0.905 | 2.864 | 0.11 |
|  | BBB leak. slope estimate | 0.941 | 0.615 | 1.441 | 0.78 |
| Normal appearing white matter | Age (unit = 5 years) | 1.060 | 0.919 | 1.223 | 0.43 |
|  | Fazekas WMH score | 1.235 | 1.036 | 1.473 | 0.019 |
|  | Mean arterial pressure | 0.987 | 0.968 | 1.006 | 0.17 |
|  | Hypertension diagnosis | 1.422 | 0.763 | 2.65 | 0.27 |
|  | Pulse pressure | 0.991 | 0.977 | 1.006 | 0.25 |
|  | Lacunar v cortical | 0.871 | 0.517 | 1.469 | 0.61 |
|  | Smoker | 1.606 | 0.904 | 2.853 | 0.11 |
|  | BBB leak. slope estimate | 0.759 | 0.441 | 1.306 | 0.32 |
| CSF | Age (unit = 5 years) | 1.042 | 0.902 | 1.205 | 0.58 |
|  | Fazekas WMH score | 1.22 | 1.023 | 1.455 | 0.027 |
|  | Mean arterial pressure | 0.986 | 0.967 | 1.005 | 0.14 |
|  | Hypertension diagnosis | 1.342 | 0.729 | 2.471 | 0.34 |
|  | Pulse pressure | 0.99 | 0.975 | 1.005 | 0.18 |
|  | Lacunar v cortical | 0.878 | 0.522 | 1.479 | 0.63 |
|  | Smoker | 1.579 | 0.888 | 2.807 | 0.12 |
|  | BBB leak. slope estimate | 1.103 | 0.998 | 1.219 | 0.056 |
| White matter hyperintensities | Age (unit = 5 years) | 1.064 | 0.922 | 1.227 | 0.40 |
|  | Fazekas WMH score | 1.234 | 1.029 | 1.48 | 0.023 |
|  | Mean arterial pressure | 0.987 | 0.968 | 1.006 | 0.18 |
|  | Hypertension diagnosis | 1.36 | 0.734 | 2.52 | 0.33 |
|  | Pulse pressure | 0.991 | 0.977 | 1.006 | 0.25 |
|  | Lacunar v cortical | 0.898 | 0.535 | 1.508 | 0.68 |
|  | Smoker | 1.594 | 0.896 | 2.835 | 0.11 |
|  | BBB leak. slope estimate | 0.921 | 0.617 | 1.374 | 0.69 |
| Index stroke lesion | Age (unit = 5 years) | 1.102 | 0.928 | 1.307 | 0.27 |
|  | Fazekas WMH score | 1.287 | 1.043 | 1.589 | 0.019 |
|  | Mean arterial pressure | 0.992 | 0.969 | 1.015 | 0.49 |
|  | Hypertension diagnosis | 1.321 | 0.585 | 2.982 | 0.50 |
|  | Pulse pressure | 0.985 | 0.968 | 1.002 | 0.086 |
|  | Lacunar v cortical | 1.052 | 0.551 | 2.009 | 0.88 |
|  | Smoker | 1.803 | 0.903 | 3.6 | 0.095 |
|  | BBB leak. slope estimate | 1.143 | 0.903 | 1.446 | 0.27 |
| Old (ie pre-existing) stroke lesion | Age (unit = 5 years) | 1.127 | 0.906 | 1.403 | 0.28 |
|  | Fazekas WMH score | 1.333 | 1.013 | 1.753 | 0.040 |
|  | Mean arterial pressure | 0.988 | 0.958 | 1.02 | 0.47 |
|  | Hypertension diagnosis | 1.21 | 0.469 | 3.12 | 0.69 |
|  | Pulse pressure | 0.987 | 0.964 | 1.01 | 0.26 |
|  | Lacunar v cortical | 0.819 | 0.344 | 1.95 | 0.65 |
|  | Smoker | 1.327 | 0.527 | 3.345 | 0.55 |
|  | BBB leak. slope estimate | 0.947 | 0.64 | 1.403 | 0.79 |

**Online Figure 1.** CONSORT Diagram recruitment and follow-up

Unable to have repeat MRI (declined, moved away, too unwell, deceased)21

Structural brain MRI180

New infarct on MRI 19

Recurrent stroke/TIA/new

lesion on scan 33

**1 year after stroke:**

Clinical follow-up 201

Cognitive testing139

Recurrent stroke 16

Recurrent TIA 5

Dependent (mRS 3-5) 31

Dead 3

Complete BBB MRI for analysis201

Diagnosis not stroke 71

Unable to have MR 39

Ineligible for other reason 53

Declined to participate 44

Enrolled in study, had clinical and diagnostic MRI264

**1-3 months after stroke:**

Attended for BBB MRI 208

Cognitive testing147

DTI MRI incomplete 2

BBB MRI data incomplete 7 (poor contrast injection, patient movement, technical fault)

Structural MRI incomplete 1

**Presentation with mild stroke**: Referred for consideration471

**Online Figure 2.** BBB leakage (top left, Pby slope) , mean diffusivity (top right, MD), fractional anisotropy (bottom left, FA) and T1 (bottom right, ms) in WMH and in incremental 2-voxel wide contours (each contour approximately 2mm wide) from the WMH edge progressively distal into normal appearing white matter, adjusted for age.


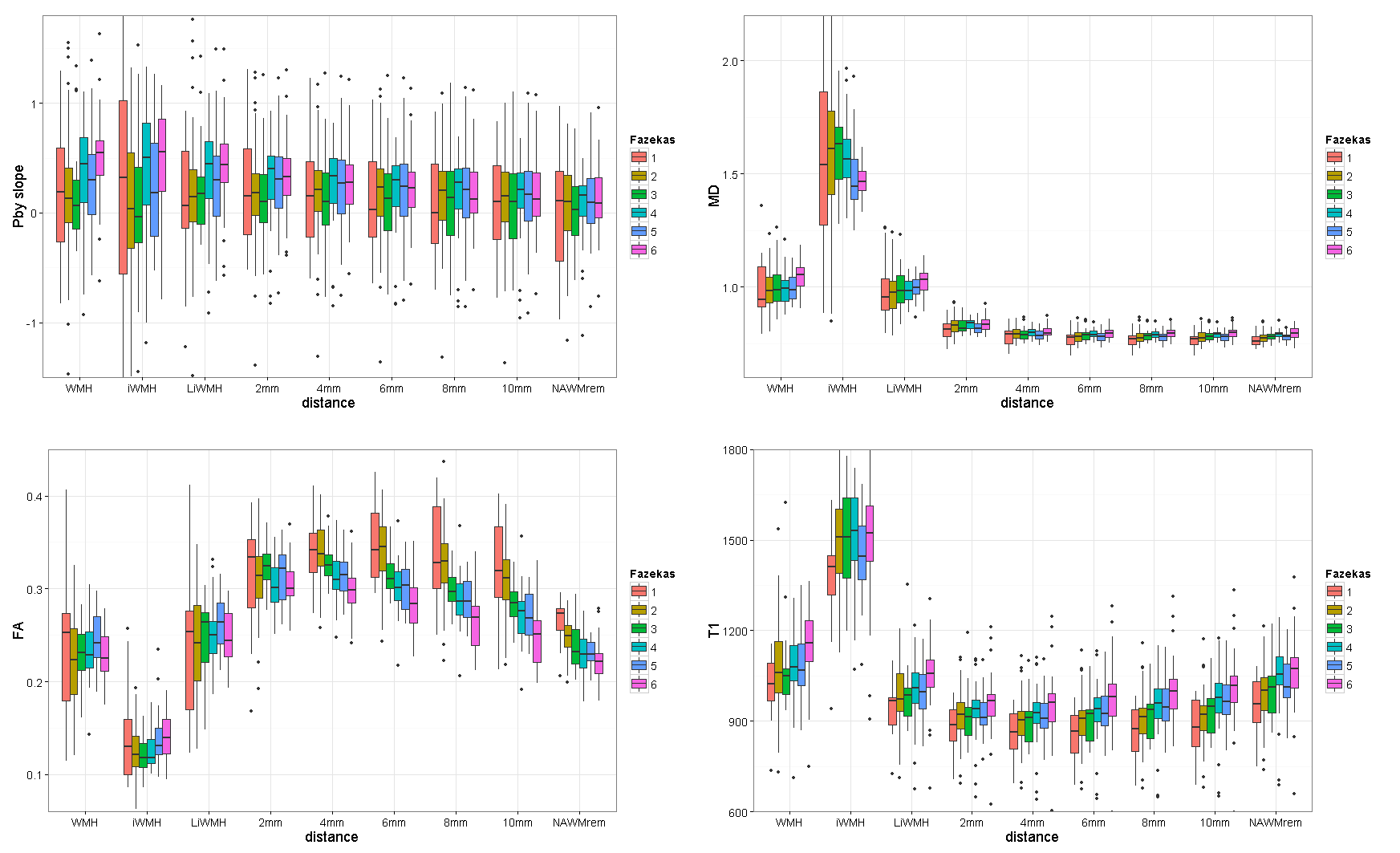


References

[1] Bamford, J., Sandercock, P., Dennis, M., Burn, J., and Warlow, C. Classification and natural history of clinically identifiable subtypes of cerebral infarction. Lancet 1991;337:1521-1526.

[2] Mioshi, E., Dawson, K., Mitchell, J., Arnold, R., and Hodges, J. R. The Addenbrooke's Cognitive Examination Revised (ACE-R): a brief cognitive test battery for dementia screening. Int J Geriatr Psychiatry 2006;21:1078-1085.

[3] Nelson, H. E. and McKenna, P. The use of current reading ability in the assessment of dementia. Br J Soc Clin Psychol 1975;14:259-267.

[4] McGurn, B., Deary, I. J., and Starr, J. M. Childhood cognitive ability and risk of late-onset Alzheimer and vascular dementia. Neurology 2008;71:1051-1056.

[5] Bamford, J., Sandercock, P., Dennis, M., Burn, J., and Warlow, C. A prospective study of acute cerebrovascular disease in the community: the Oxfordshire Community Stroke Project - 1981-86. 2. Incidence, case fatality rates and overall outcome at one year of cerebral infarction, primary intracerebral and subarachnoid haemorrhage. J Neurol Neurosurg Psychiatry 1990;53:16-22.

[6] Dennis, M., Mead, G., Doubal, F., and Graham, C. Determining the modified Rankin score after stroke by postal and telephone questionnaires. Stroke 2012;43:851-853.

[7] Wardlaw, J. M., Smith, E. E., Biessels, G. J., Cordonnier, C., Fazekas, F., Frayne, R. et al. Neuroimaging standards for research into small vessel disease and its contribution to ageing and neurodegeneration: a united approach. Lancet Neurol 2013;12:822-838.

[8] Valdés Hernández, M., Armitage, P., Thrippleton, M. J., Chappell, F., Sandeman, E., Munoz Maniega, S. et al. Rationale, design and methodology of the image analysis protocol for studies of patients with cerebral small vessel disease and mild stroke. Brain Behav 2015;DOI: 10.1002/brb3.415:

[9] Wardlaw, J. M., Bastin, M. E., Valdes Hernandez, M. C., Munoz Maniega, S., Royle, N. A., Morris, Z. et al. Brain aging, cognition in youth and old age and vascular disease in the Lothian Birth Cohort 1936: rationale, design and methodology of the imaging protocol. Int J Stroke 2011;6:547-559.

[10] Fazekas, F., Chawluk, J. B., Alavi, A., Hurtig, H. I., and Zimmerman, R. A. MR signal abnormalities at 1.5T in Alzheimer's dementia and normal aging. AJR Am J Roentgenol 1987;149:351-356.

[11] The IST-3 Collaborative Group. Association between brain imaging signs, early and late outcomes, and response to intravenous alteplase after acute ischaemic stroke in the third International Stroke Trial (IST-3): secondary analysis of a randomised controlled trial. Lancet Neurol 2015;14:485-496.

[12] Valdes Hernandez, M. C., Ferguson, K. J., Chappell, F. M., and Wardlaw, J. M. New multispectral MRI data fusion technique for white matter lesion segmentation: method and comparison with thresholding in FLAIR images. Eur Radiol 2010;20:1684-1691.

[13] Armitage, P. A., Farrall, A. J., Carpenter, T. K., Doubal, F. N., and Wardlaw, J. M. Use of dynamic contrast-enhanced MRI to measure subtle blood-brain barrier abnormalities. Magn Reson Imaging 2011;29:305-314.

[14] Heye, A. K., Culling, R. D., Valdes Hernandez, M. C., Thrippleton, M. J., and Wardlaw, J. M. Assessment of blood–brain barrier disruption using dynamic contrast-enhanced MRI. A systematic review. Neuroimage Clin 2014;6:262-274.

[15] Montagne, A., Barnes, S. R., Sweeney, M. D., Halliday, M. R., Sagare, A. P., Zhao, Z. et al. Blood-brain barrier breakdown in the aging human hippocampus. Neuron 2015;85:296-302.

[16] Heye, A. K., Thrippleton, M. J., Armitage, P. A., Valdes Hernandez, M. C., Makin, S. D., Glatz, A. et al. Tracer kinetic modelling for DCE-MRI quantification of subtle blood-brain barrier permeability. Neuroimage 2016;125:446-455.

[17] Brookes, J. A., Redpath, T. W., Gilbert, F. J., Murray, A. D., and Staff, R. T. Accuracy of T1 measurement in dynamic contrast-enhanced breast MRI using two- and three-dimensional variable flip angle fast low-angle shot. J Magn Reson Imaging 1999;9:163-171.

[18] Heye, A. K., Thrippleton, M. J., Armitage, P. A., Valdes Hernandez, M. C., Makin, S. D., Glatz, A. et al. Tracer kinetic modelling for DCE-MRI quantification of subtle blood-brain barrier permeability. Neuroimage 2016;125:446-455.

[19] Barnes, S. R., Ng, T. S., Montagne, A., Law, M., Zlokovic, B. V., and Jacobs, R. E. Optimal acquisition and modeling parameters for accurate assessment of low K blood-brain barrier permeability using dynamic contrast-enhanced MRI. Magn Reson Med 2015;10.1002/mrm.25793:

[20] Heye, A. K., Thrippleton, M. J., Chappell, F. M., Valdes Hernandez, M. C., Armitage, P. A., Makin, S. D. et al. Blood pressure and sodium: association with MRI markers in cerebral small vessel disease. J Cereb Blood Flow Metab 2016;36:264-274.

[21] Munoz Maniega, S., Valdes Hernandez, M., Clayden, J. D., Royle, N. A., Murray, C., Morris, Z. et al. White matter hyperintensities and normal-appearing white matter integrity in the aging brain. Neurobiol Aging 2015;36:-909.

[22] Munoz Maniega, S., Chappell, F. M., Valdes Hernandez, M. C., Armitage, P. A., Makin, S. D., Heye, A. K. et al. Integrity of normal-appearing white matter: influence of age, visible lesion burden and hypertension in patients with small vessel disease. J Cereb Blood Flow Metab 2016;in press:
